# Elemene Augments the Effects of Anti‐PD‐1 Immunotherapy on Hepatocellular Carcinoma by Regulating the miR‐130a‐5p/SPP/MHC‐I Axis

**DOI:** 10.1002/advs.202511887

**Published:** 2026-01-12

**Authors:** Menglan Wang, Mengqing Sun, Heng Dong, Xiaoting Zhang, Jingbo Zhang, Zhengguo Zhang, Mengjie Ni, Lina Li, Yunxin Pei, Xiaoyu Chen, Qian Li, Fangtian Bu, Jiacheng Huang, Liangyu Jiang, Zhuting Fang, Xuliang Chen, Jianxiang Chen, Yiting Qiao, Tian Xie

**Affiliations:** ^1^ Zhejiang Provincial Key Laboratory of Anti‐Cancer Chinese Medicines and Natural Medicines School of Pharmacy Hangzhou Normal University Department of Hepatology the Affiliated Hospital of Hangzhou Normal University Hangzhou 311121 China; ^2^ Division of Hepatobiliary and Pancreatic Surgery Department of Surgery The First Affiliated Hospital, Zhejiang University School of Medicine NHC Key Laboratory of Combined Multi‐organ Transplantation Key Laboratory of Organ Transplantation Key Laboratory of the Diagnosis and Treatment of Organ Transplantation Research Unit of Collaborative Diagnosis and Treatment for Hepatobiliary and Pancreatic Cancer Chinese Academy of Medical Science (2019RU019) Hangzhou 310003 China; ^3^ Faculty of Chinese Medicine and State Key Laboratory of Quality Research in Chinese Medicine Macau University of Science and Technology Macao 999078 China; ^4^ Department of Oncology and Vascular Intervention Clinical Oncology School of Fujian Medical University Fujan Cancer Hospital Fuzhou Fujian 350001 China; ^5^ Key Laboratory of Artificial Organs and Computational Medicine in Zhejiang Province Shulan (Hangzhou) Hospital Shulan International Medical College Zhejiang Shuren University Hangzhou 310015 China

**Keywords:** antigen presentation, cytotoxic T lymphocytes, immune checkpoint inhibitors, organoids, tumor immune microenvironment

## Abstract

Immune checkpoint inhibitors, particularly PD‐1 inhibitors, constitute the cornerstone of first‐line treatment for hepatocellular carcinoma (HCC). However, suboptimal overall response rates persist alongside dual challenges: immune‐related toxicities and immunosuppressive tumor microenvironment. Combination therapies represent a pivotal strategy to overcome these limitations. In recent years, traditional Chinese medicine has gained significant attention. Elemene, a small‐molecule compound derived from *Curcuma wenyujin*, has garnered attention for its immunomodulatory potential and demonstrates clinical efficacy in combination therapies. Nevertheless, its synergistic mechanisms with immunotherapy remain incompletely characterized. This study demonstrates for the first time that elemene modulates the miR‐130a‐5p/SPP/MHC‐I axis, resulting in an enhanced diversity and abundance of antigen/MHC‐I complexes on the surface of HCC cells. This mechanism promotes the recognition and elimination of HCC cells by cytotoxic T lymphocytes, thereby augmenting the antitumor immune efficacy of PD‐1. Moreover, the functional significance of the miR‐130a‐5p/SPP/MHC‐I axis in modulating the tumor immune microenvironment is systematically validated through in vitro and in vivo HCC models, as well as in clinical patient specimens. These findings underscore the potential of combining elemene with anti‐PD‐1 therapy as a safe and effective treatment strategy for HCC, offering significant translational promise for improving patient outcomes.

## Graphical abstract

Elemene increases SPP expression by competitively binding with miR‐130a‐5p to suppress SPP mRNA degradation. This led to more antigen/MHC‐I complexes being expressed on the cell surface, which consequently facilitated the recognition and killing of HCC cells by CTLs and enhancing the antitumor immune efficacy of anti‐PD‐1.

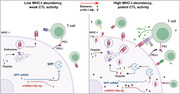

## 1 Introduction

Hepatocellular carcinoma (HCC) is the third leading cause of cancer‐related death worldwide.^[^ ^1^, ^2^ ^]^ Owing to the lack of typical symptoms, more than two‐thirds of HCC patients have progressed to advanced stages at the time of diagnosis, and the 5‐year survival rate is less than 15%.^[^ ^3^, ^4^ ^]^ Systemic therapies, including immune checkpoint inhibitors (ICIs), have been applied to patients with inoperable advanced HCC.^[^ ^5^, ^6^ ^]^ In general, ICIs reactivate anticancer immune responses by blocking immune‐inhibitory crosstalk between cancer cells and cytotoxic T lymphocytes (CTLs) to potently eliminate tumor cells by remodeling the tumor immune microenvironment (TIME).^[^ ^7^ ^]^ However, a highly immunosuppressive TIME is established in HCC through multiple mechanisms, resulting in only 18%–20% of HCC patients benefit from ICI monotherapy.^[^ ^8^, ^9^ ^]^ Clinical studies have shown that the combination of anti‐PD‐1 and lenvatinib can significantly improve the overall response rate of HCC patients to 55.4%, suggesting that combination therapy can improve the therapeutic efficacy of HCC immunotherapy.^[^ ^10^, ^11^ ^]^ Nevertheless, almost half of the patients remain nonresponsive, and ICI treatment carries the risk of serious adverse reactions, such as myocarditis.^[^ ^12^ ^]^ Therefore, it is imperative to develop novel agents that synergize with existing immunotherapies, overcome immune resistance, and mitigate toxicity during the journey of combating HCC.

Traditional Chinese Medicine (TCM) and its bioactive compounds represent promising resources for novel drug discovery.^[^ ^13^, ^14^ ^]^ *Curcuma wenyujin* (*C. wenyujin*) is a TCM used in various conditions, such as liver disease, thrombosis, pain, and arthritis.^[^ ^15^ ^]^ Elemene(C_15_H_24_), a small terpenoid extracted from *C. wenyujin*, is recognized as an important plant‐derived antitumor agent.^[^ ^16^, ^17^ ^]^ Modern pharmacological studies have identified elemene as the primary bioactive compound responsible for the hepatoprotective properties of *C. wenyujin*, and it has achieved a good synergistic effect as an adjuvant drug in clinical combination therapy.^[^ ^18^ ^]^ Chemotherapy combined with elemene can significantly improve survival rate and tumor response, reduce toxicity, and reverse drug resistance.^[^ ^19^, ^20^ ^]^ A preliminary evaluation conducted by our team assessed the safety of elemene for the treatment of malignant tumors, and confirmed that elemene could improve the antitumor efficacy of the original treatment with no obvious toxic side effects (Table S1 and S2, Supporting Information). Moreover, the U.S. Food and Drug Administration (FDA) has granted approval for elemene to advance to Phase 2‐3 sequential clinical trials under orphan drug designation (IND NUMBER ASSIGNED:162022). However, the influence of elemene on T‐cell‐oriented ICI‐mediated immunotherapy has not been thoroughly evaluated, especially in HCC. Given its established safety and adjuvant benefits, we propose that elemene may potentiate anti‐PD‐1 therapy in HCC. This study, therefore, investigated the therapeutic synergy of elemene combined with anti‐PD‐1 immunotherapy to address unmet needs in HCC treatment.

A key mechanism underlying HCC immune evasion involves impaired antigen presentation, specifically reduced cell surface expression of antigen/MHC‐I complexes.^[^ ^21^, ^22^ ^]^ During hepatocarcinogenesis, structural genes essential for MHC‐I synthesis are frequently inactivated, and patients with low MHC‐I expression exhibit greater resistance to immunotherapy.^[^ ^23^, ^24^ ^]^ Consequently, augmenting antigen/MHC‐I complex expression represents a promising strategy to overcome immune evasion. Signal peptide peptidase (SPP), encoded by the HM13 (SPP) gene, is a crucial intramembrane aspartyl protease that cleaves signal peptide into short immunogenic peptides. The primary function of SPP is to cleave the remnant signal peptides derived from newly synthesized proteins in the Endoplasmic Reticulum (ER) membrane. A key consequence of this activity is that SPP processes these signal peptides into short peptides that are subsequently transported into the ER lumen. Previous experiments have demonstrated that these peptides can be directly loaded onto MHC‐I molecules for antigen presentation.^[^ ^25^, ^26^ ^]^ Compared with empty complexes, the embedding of antigens into MHC‐I complexes not only endows immunogenicity but also extends the half‐life of the MHC‐I complex on the cell surface.^[^ ^27^ ^]^ Therefore, SPP might play a key role in the antigen/MHC‐I complex‐mediated recognition of tumor cells by the immune system. In a high‐throughput screen for HIV resistance‐related genes in CD4^+^ T lymphocytes, SPP was identified as one of the key genes mediating interferon production upon HIV infection, indicating its involvement in T‐cell‐mediated immune responses.^[^ ^28^ ^]^ However, the involvement of SPP in TIME remodeling and immunotherapy has not been intensively investigated in HCC.

In this study, we found that elemene significantly enhances the therapeutic effects of anti‐PD‐1 immunotherapy without obvious systemic toxicity in murine xenograft and orthotopic HCC models. A mechanistic study revealed that the combination of elemene and anti‐PD‐1 robustly enhanced the intratumoral infiltration, clonal expansion, and functions of CTLs. Further mechanistic studies revealed that elemene upregulates SPP expression by competitively binding with miR‐130a‐5p to suppress the degradation of SPP mRNA. This led to the expression of more antigen/MHC‐I complexes on the surface of HCC cells, thereby facilitating the recognition and killing efficacy of HCC cells by CTLs. Moreover, the importance of the miR‐130a‐5p/SPP/MHC‐I axis in the TIME was validated in both in vitro and in vivo HCC models as well as in clinical specimens. Overall, our findings demonstrate that elemene potentiates anti‐PD‐1 immunotherapy efficacy on HCC via the miR‐130a‐5p/SPP/MHC‐I axis. This combination could represent a promising therapeutic approach for HCC, offering a potential strategy to overcome resistance to immunotherapy.

## 2 Results

### 2.1 Elemene Synergistically Enhanced the Anti‐HCC Effects of Anti‐PD‐1

**Figure 1:**
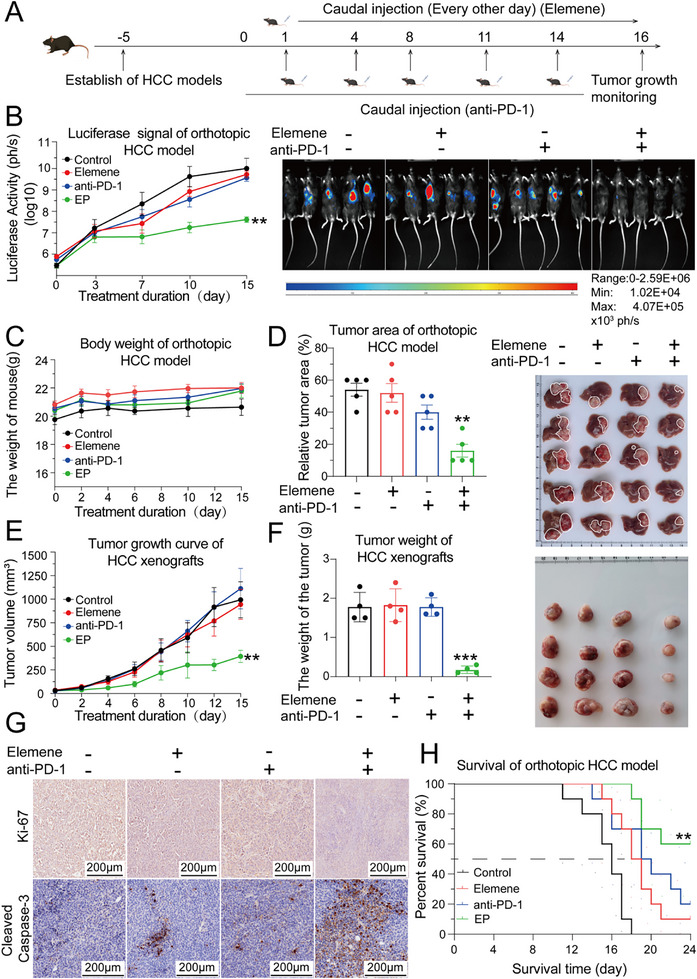
EP therapy significantly inhibited tumor growth in murine orthotopic and subcutaneous xenograft HCC models. A) Experimental schema for establishing the murine HCC models. B) Curves of the signal intensity of the in situ luminescence images and a representative luminescence photograph of mice bearing orthotopic HCC tumors after treatment with elemene or anti‐PD‐1 antibody alone or EP therapy. C) Curves of the body weights of the orthotopic HCC model after various treatment regimens. The data are presented as the means ± SEM. D) Histogram showing the tumor areas and a photograph of the livers resected from the orthotopic HCC models. *n* =5. The data are presented as the means ± SEM. E) Tumor growth curves of the subcutaneous xenograft HCC model. F) Histogram of the tumor weights of the subcutaneous xenograft HCC model treated with the indicated regimens and photograph of tumors resected from the subcutaneous xenograft HCC model. *n*=4. The data are presented as the means ± SEM. G) Immunohistochemical analyses of Ki‐67 and cleaved caspase‐3 in the tumor tissues of the orthotopic HCC models. Scale bar = 200 µm. H) Kaplan–Meier survival curves of the orthotopic HCC model treated with various regimens. A log‐rank analysis was performed to compare each treatment group to the control group. *n* = 10. ^**^ *p* < 0.01, ^***^ *p* < 0.001.

To evaluate the combined therapeutic efficacy of elemene plus anti‐PD‐1, two murine HCC models were established in C57BL/6J mice, which were treated with either elemene, anti‐PD‐1, or a combination of elemene and anti‐PD‐1 (abbreviated as EP therapy hereafter). Elemene was administered via tail vein injection every other day when the tumor became palpable, and the anti‐PD‐1 was administered intravenously three times a week (**Figure** **1**A). As shown by the signal intensities upon in situ luciferase imaging and photographs of resected livers from orthotopic HCC models, monotherapy with elemene or the anti‐PD‐1 led to a marginal reduction in the speed of HCC progression, whereas EP therapy exhibited a remarkable anti‐HCC effect compared to each monotherapy group (Figure 1B–D). No significant changes had been noted in total body weight, serum indexes such as ALT, or tissue morphology of the liver, spleen, or kidney in any treatment group compared to the control group, indicating that EP therapy had low systemic toxicity (Figure S1A–G, Supporting Information). Similarly, both the tumor volume growth curve and image of the resected tumors demonstrated that EP therapy also exhibited synergistic anti‐HCC effects in the subcutaneous HCC xenograft model (Figure 1E,F). Immunohistochemical (IHC) analysis of HCC samples from both HCC models revealed a remarkable reduction in Ki‐67 positive regions and an increase in cleaved caspase‐3 positive regions following EP therapy (Figure 1G). Moreover, a survival experiment was conducted to elucidate the therapeutic effects of EP therapy in the murine orthotopic HCC model, and the results revealed that EP therapy significantly prolonged the survival time compared with that of elemene or anti‐PD‐1 monotherapy (Figure 1H).

In conclusion, EP therapy synergistically inhibited HCC progression without causing obvious systemic toxicity.

### 2.2 The Combination of Elemene and Anti‐PD‐1 Significantly Potentiated the Intratumoral Infiltration of Functional CTLs

**Figure 2:**
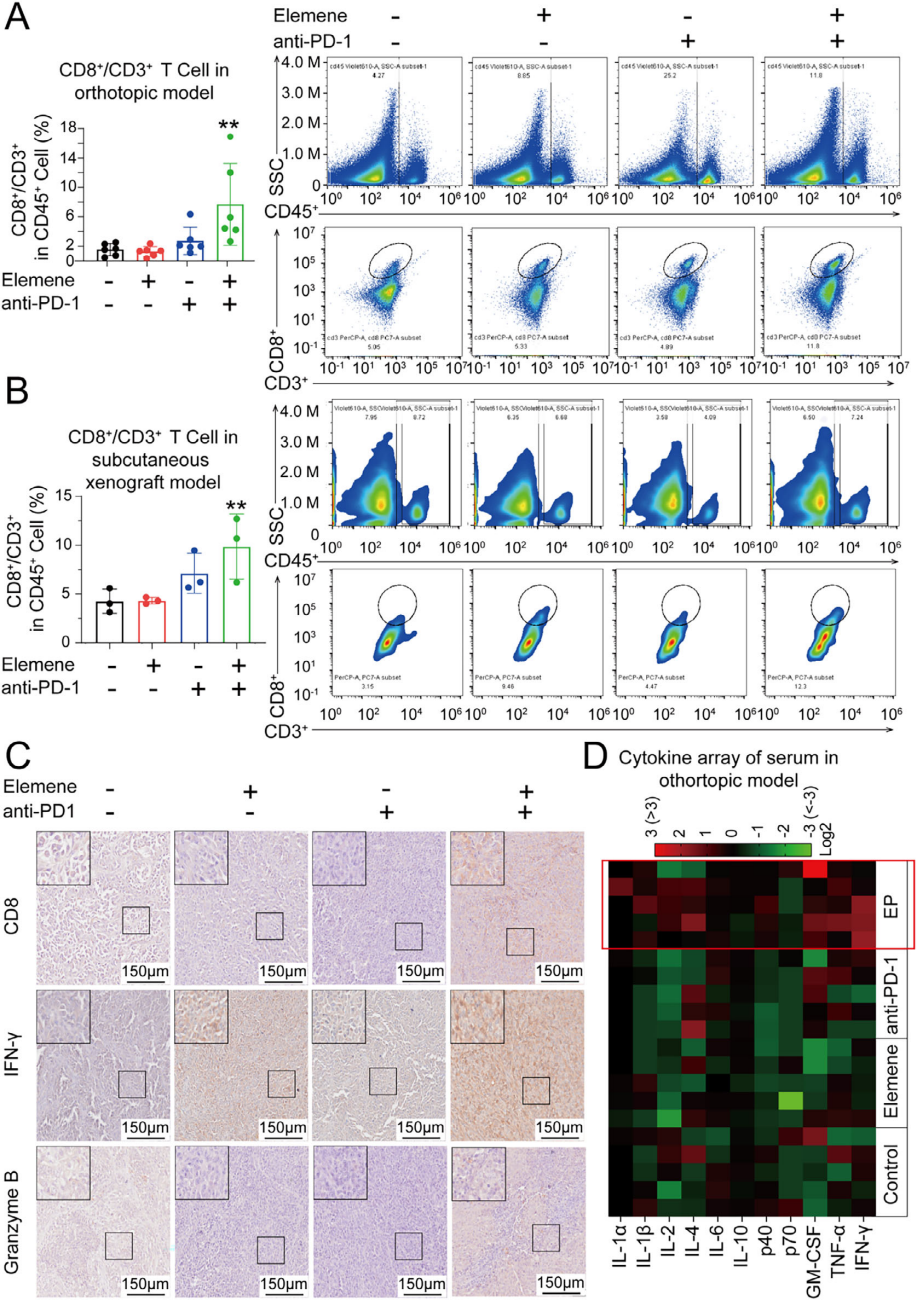
The intratumoral infiltration of Cytotoxic T Lymphocyte (CTL) was enhanced in both the orthotopic and subcutaneous xenograft HCC models after EP therapy. A) Histogram of the percentage and representative images of flow cytometry analysis on CD8^+^/CD3^+^ T expression of CD45^+^ lymphocytes in tumor tissues of orthotopic HCC model. *n* = 6. The data are presented as the means ± SEM. B) Histogram of the percentage and representative images of flow cytometry analysis on CD8^+^/CD3^+^ T expression of CD45^+^ lymphocytes in tumor tissues of subcutaneous xenograft HCC model. *n* = 3. The data are presented as the means ± SEM. C) Representative immunohistochemistry (IHC) images of CD8, IFN‐γ, and Granzyme B in the tumor tissues of the orthotopic HCC model. Scale bar = 150 µm. D) Heatmap showing the serum levels of proinflammatory cytokines in the peripheral blood of the orthotopic HCC model after the indicated treatments. *n* = 5. ^**^ *p* < 0.01.

Given that elemene has been reported to modulate immune responses, and considering that the number and functional status of CTLs are key factors determining the therapeutic efficacy of immunotherapy, and we speculated that EP therapy might favorably influence CTLs.^[^ ^29^, ^30^ ^]^ Therefore, intratumoral lymphocytes were analyzed via flow cytometry, and the results revealed that the proportion of CD8^+^ T, CD4^+^ T cells among CD45^+^CD3^+^ cells is significantly increased after EP therapy in both the subcutaneous xenograft model and the orthotopic HCC model compared with each monotherapy group (**Figure** **2**A,B; Figure S2A,B, Supporting Information). These results demonstrated that the combination of elemene and the anti‐PD‐1 synergistically increased the number of CTLs in HCC tissues. Consistent with previous observations, the IHC results revealed that the HCC tissues from mice treated with EP therapy exhibited the highest signal intensities of CD8, IFN‐γ, and Granzyme B, three intratumoral CTL markers, indicating that the number and cytotoxic functions of CTLs were synergistically improved by EP therapy compared with each monotherapy (Figure 2C). Moreover, peripheral blood samples from the orthotopic HCC models after indicated therapies were subjected to a cytokine array, and the results demonstrated that the levels of proinflammatory cytokines, such as GM‐CSF, TNF‐α, and IFN‐γ, markedly increased after EP therapy, indicating rigorous remodeling of the overall immune status toward immune activation after EP therapy (Figure 2D).

In short, the combination of elemene and anti‐PD‐1 significantly increased the intratumoral infiltration and functions of CTLs.

### 2.3 Elemene Promoted the Abundance and Diversity of MHC‐I/Peptide Complexes on the Surface of HCC Cells by Upregulating SPP Expression

**Figure 3:**
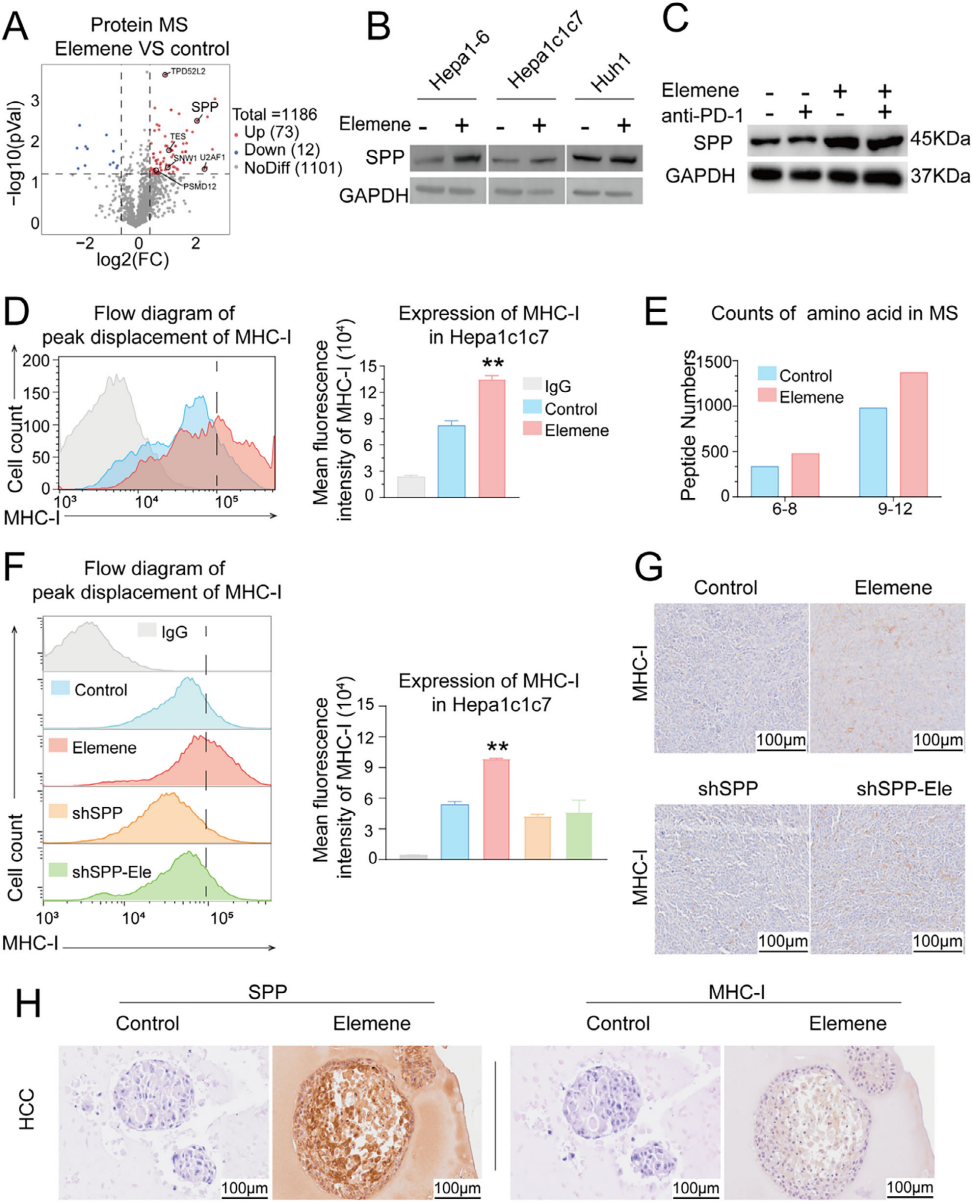
Elemene increased the abundance of MHC‐I/peptide complexes on the surface of HCC cells by increasing SPP expression. A) Volcano plot showing the significantly differentially expressed proteins in Hepa1c1c7 cells after elemene treatment determined via quantitative proteomics. (|Log2FC| > 0.5, *p < 0.05*). B) Western blots showing SPP protein levels in 3 HCC cell lines treated with elemene. C) Western blots showing SPP protein levels in the HCC orthotopic model. D) Representative flow cytometry histogram and quantitative analysis of the mean fluorescence intensity showing the MHC‐I expression level in Hepa1c1c7 cells after elemene treatment. *n*=3. The data are presented as the means ± SD. E) Histogram showing the number of unique peptides in the immunoprecipitants of MHC‐I antibodies in Hepa1c1c7 cells after elemene treatment, as determined by mass spectrometry analysis. F) Representative flow cytometry histogram and quantitative analysis of the mean fluorescence intensity showing the change in MHC‐I expression after elemene treatment in Hepa1c1c7 cells transfected with shscramble or shSPP. *n* = 3. The data are presented as the means ± SD. G) Representative IHC images showing MHC‐I expression in the tumor tissues of the orthotopic HCC model. Scale bar = 100 µm. H) Representative IHC images showing the expression of SPP and MHC‐I in patient‐derived organoids of HCC treated with elemene. Scale bar = 100 µm. ^**^ *p* < 0.01.

We hypothesized that anti‐PD1 therapy serves as primary immunotherapy in these models, and that elemene functions as an adjuvant immunomodulator to reshape the tumor microenvironment (TME), thereby enhancing CTL activity. Since tumor parenchymal cells are the most abundant cell type in the HCC microenvironment, we speculated that elemene may influence key proteins associated with the immunogenic characteristics of HCC cells. To test this hypothesis, a quantitative proteomics assay was performed via protein mass spectrometry (MS) on Huh1, a human HCC cell line, and Hepa1c1c7, a mouse HCC cell line. Interestingly, SPP was significantly upregulated at the protein level after elemene treatment in both HCC cell lines (**Figure** **3**A; Figure S3A, Supporting Information). This observation was validated via western blot (Figure 3B,C; Figure S3B, Supporting Information) and quantitative polymerase chain reaction (qPCR) (Figure S4A–C, Supporting Information) in other HCC cell lines as well as the murine orthotopic HCC model with various combination treatments. The results demonstrated that the expression of SPP was potently induced by elemene in HCC cells.

Existing evidence has shown that SPP, an endoplasmic reticulum protease, plays a critical role in antigen processing and presentation, and silencing SPP leads to a general decrease in the number of surface MHC‐I/peptide complexes.^[^ ^31^ ^]^ Therefore, we speculated that elemene treatment, which induced SPP expression, could increase the abundance of surface MHC‐I/peptide complexes in HCC cells. To test this hypothesis, MHC‐I levels were measured by flow cytometry, and the results showed that elemene could induce an increase in surface MHC‐I levels, as indicated by the rightward shift in the fluorescent signal peak of elemene‐treated cells (Figure 3D; Figure S3C, Supporting Information). Additionally, antigen diversity was analyzed by MS of the immune precipitates of MHC‐I antibodies after elemene treatment, and the results revealed that elemene treatment markedly increased antigen diversity, as indicated by the number of unique short peptides (≤12 amino acid residues) (Figure 3E). The above results suggested that elemene could increase the abundance and diversity of MHC‐I/peptide complexes on the surface of HCC cells.

To validate that SPP was the key mediator in the elemene‐induced increase in MHC‐I/peptide complex abundance, surface MHC‐I levels were analyzed via flow cytometry after silencing SPP via shRNA in HCC cells, and the results revealed that the elemene‐induced rightward shift in the MHC‐I fluorescent signal peak was greatly diminished in cells pretreated with SPP shRNA (Figure 3F; Figure S3D, Supporting Information). Importantly, IHC analysis in the orthotopic HCC model confirmed that SPP knockdown effectively abolished the elemene‐induced upregulation of MHC‐I expression (Figure 3G). These results demonstrated that the elemene‐induced increase in the number of surface MHC‐I/peptide complexes was mainly mediated by SPP in HCC cells.

To investigate whether elemene can induce the SPP/MHC‐I axis in clinical specimens of HCC and other types of cancer cells, patient‐derived organoids of various cancer types, including HCC, colorectal cancer (CRC), intrahepatic cholangiocarcinoma (ICC), and lung cancer (LC), were treated with elemene. IHC analysis of SPP and MHC‐I in these organoids revealed that the expression levels of SPP and MHC‐I generally increased after the treatment of elemene, indicating the universality of elemene induction of the SPP/MHC‐I axis in solid tumors in clinical practice (Figure 3H; Figure S3E, Supporting Information).

The above results demonstrated that elemene enhances the expression of SPP, which subsequently increases the abundance and diversity of surface MHC‐I/peptide complexes in HCC cells.

### 2.4 Elemene Potentiated CTL Clonal Diversity and Expansion via the SPP/MHC‐I Axis

Recognition between the MHC‐I/peptide complex and the structurally complementary T‐cell receptor (TCR) is the initial step in the activation of tumor‐specific CTLs, and a positive correlation between the abundance of membrane MHC‐I/peptide complexes and intratumoral CTLs has been demonstrated in various types of solid tumors.^[^ ^32^, ^33^ ^]^ Our aforementioned data revealed that SPP is a key mediator of the elemene‐induced increase in MHC‐I in HCC cells, but the relationship between intratumoral T cells and elemene‐induced SPP/MHC‐I axis activation required further validation in vivo.

**Figure 4:**
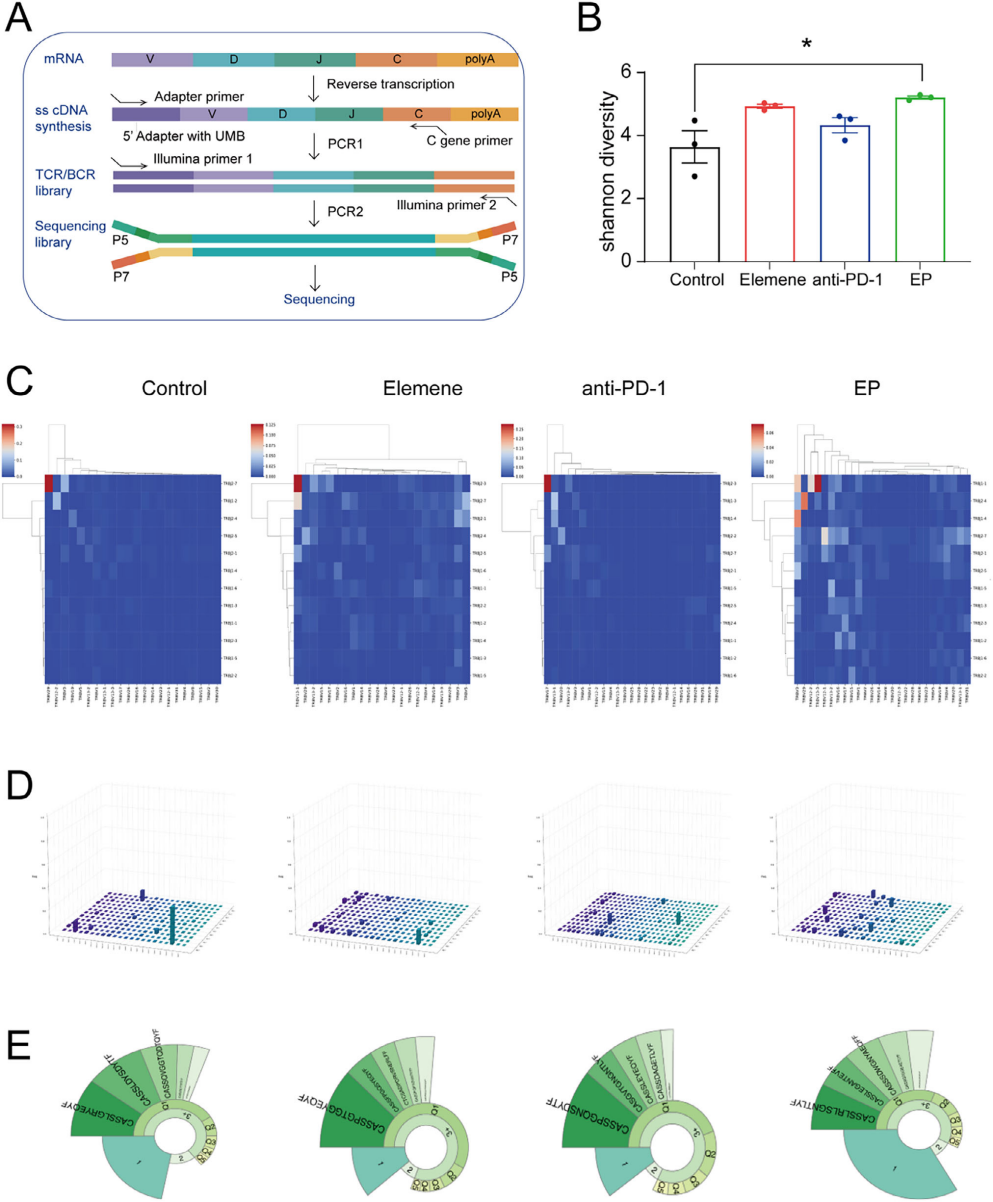
Elemene increased CTL diversity during HCC immunotherapy. A) Flowchart of T‐cell receptor (TCR) sequencing. B) Comparison of T cell clonal diversity based on Shannon Diversity Index. The Shannon diversity index was calculated from TCR sequencing data to quantitatively evaluate the diversity of the T cell repertoire across different treatment groups. *n* = 3. The data are presented as the means ± SD. C,D) Heatmaps (C) and 3D forest maps (D) showing the frequency of each TCR‐β chain V–J fragment combination in the tumor tissues of subcutaneous xenograft HCC model after the indicated treatment. The high‐, moderate‐, and low‐frequency combinations in the heatmaps are colored red, white, and blue, respectively. The frequency of each V–J fragment combination is shown as the height of the bar in the 3D forest maps. E) Snail plots showing the classification of TCR‐β chain clonal frequencies in tumor tissues of the subcutaneous xenograft HCC model after the indicated treatments. Categorizes all detected TCR sequences into "1″, "2″, and "3+" groups, representing TCR molecules detected once, twice, or more than 3 times in the TCR sequencing assay, respectively. The "1″ category consists of a large number of distinct T‐cell clones, whose TCR sequences were only detected once during the TCR sequencing assay. These low‐abundance clones are precisely the basis of clonal polymorphism. ^*^ *p* < 0.05.

Therefore, to directly assess the functional impact of this axis on the CTL repertoire, we performed T‐cell receptor (TCR) sequencing on tumors from the subcutaneous HCC xenograft models. Using a 5′ RACE protocol for unbiased amplification of the TCR‐β chain, we analyzed the composition of V, D, J, and C segments (**Figure** **4**A). Our analysis revealed that EP combination therapy significantly enhanced the diversity of the T‐cell repertoire compared to monotherapies. This was quantitatively demonstrated by a marked increase in the Shannon diversity index in the EP group (Figure 4B). The repertoire characteristics were further visualized by heatmaps and 3D forest plots, which illustrated a broader spectrum of V‐J fragment combinations in the treatment groups, particularly following EP therapy (Figure 4C,D). Furthermore, the clone frequency classification was analyzed, and the data were presented as snail plots (Figure 4E), wherein the area labeled “1” refers to “unique T cell clones with the lowest frequency”. The “1” category consists of a large number of distinct T‐cell clones, whose TCR sequences were only detected once during the TCR sequencing assay. These low‐abundance clones are precisely the basis of clonal polymorphism. The snail plots revealed that the tumors in the EP therapy group presented the highest frequency of monoclonal T cells among all groups, indicating that combination therapy increased the CTL amplification potential in HCC. In general, EP therapy resulted in greater CTL clonal diversity than anti‐PD‐1 monotherapy alone.

Taken together, the SPP/MHC‐I axis induced by elemene could increase the number and diversity of intratumoral CTLs during immunotherapy. However, the molecular mechanism of how elemene induces SPP expression requires further investigation.

### 2.5 Elemene Stabilized SPP mRNA by Antagonizing miR‐130a‐5p

**Figure 5:**
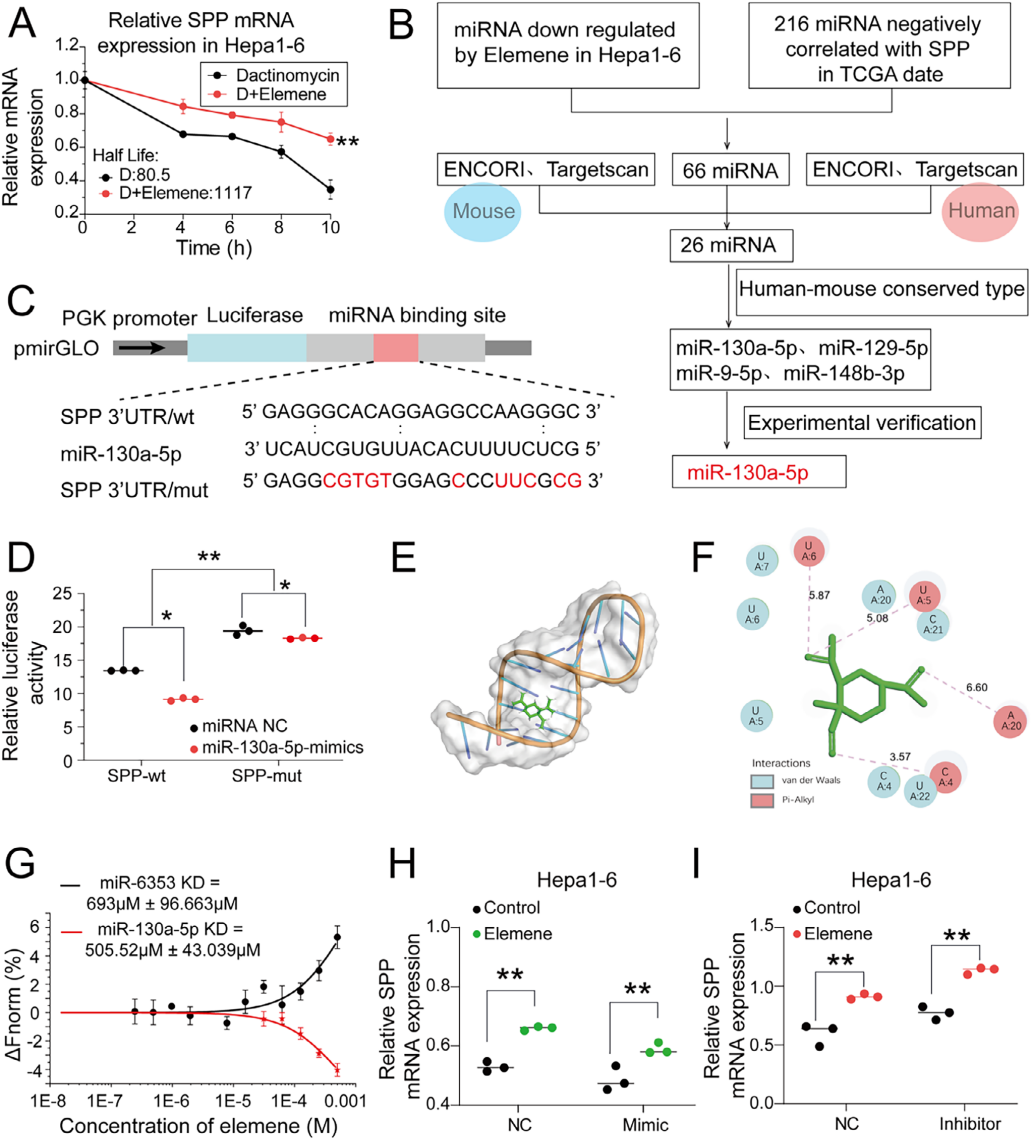
Elemene stabilized SPP mRNA by antagonizing miR‐130a. A) Degradation curves of SPP mRNA in Hepa1‐6 cells pretreated with elemene following actinomycin D treatment. Nonlinear regression curve fitting (one‐phase decay) was used to calculate the decay rates. *n* = 3. The data are presented as the means ± SEM. B) Flowchart for the identification of potential miRNAs involved in the elemene‐induced retardation of SPP mRNA degradation. C) Diagrammatic representation of the wild‐type (WT) and mutant (mut) SPP luciferase reporter plasmids. D) Relative luciferase activity produced by the SPP WT and mut plasmids in the absence or presence of the miR‐130a‐5p mimic in Hepa1‐6 cells. *n* = 3. The data are presented as the means ± SD. E,F) Molecular docking modes for miR‐130a‐5p and elemene in surface map(E) and 2D map (F). Note that the elemene molecule (green) is located at its binding site on the molecular surface of miR‐130a‐5p (gray) in surface map (E), and the binding interactions between elemene (green stick model) and the surrounding nucleic acid residues (circles) of miR‐130a‐5p are shown in 2D map (F). G) MST curves showing the affinity between elemene and miR‐130a‐5p. Dissociation constant, K_D_. H,I) Relative SPP mRNA levels in Hepa1‐6 cells pretreated with elemene in the presence or absence of the miR‐130a‐5p mimic (H) or inhibitor(I). *n* = 3. The data are presented as the means ± SD. ^*^ *p* < 0.05, ^**^ *p* < 0.01.

The stability of SPP mRNA was evaluated, and the qPCR assay revealed that under the treatment of actinomycin D, elemene retarded the degradation of SPP mRNA (**Figure** **5**A; Figure S5A, Supporting Information). miRNAs are key players that mediate mRNA degradation via complementary base pairing.^[^ ^34^ ^]^ Therefore, we speculated that elemene might interfere with the binding of miRNAs to SPP mRNA to suppress its degradation. To identify the potential miRNAs involved in this process, miRNA sequencing was performed in Hepa1‐6 cells, and 216 miRNAs were significantly downregulated after elemene treatment. Among them, the expression levels of 66 miRNAs were negatively correlated with the mRNA level of SPP based on the TCGA database. Then, base pairing prediction revealed that 26 miRNAs had the potential binding site with SPP mRNA in the ENCORI and TargetScan miRNA prediction databases. Finally, the list was narrowed down to 4 miRNAs based on conservative analysis between humans and mice (Figure 5B). Finally, miR‐130a‐5p was selected for further analysis due to its uniform downregulation following elemene treatment in multiple HCC cell lines, as confirmed by qPCR assays. (Figure S5B–D, Supporting Information). Furthermore, the HCC tissues of the mice that received elemene treatment also presented significantly reduced miR‐130a‐5p expression compared with the HCC tissues of untreated mice (Figure S5E,F, Supporting Information).

To determine whether miR‐130a‐5p directly mediated the degradation of SPP mRNA, the 3′UTR of SPP mRNA was inserted into a luciferase reporter plasmid, and the potential miR‐130a‐5p binding site was identified and deleted to construct a mutant reporter (Figure 5C). As shown by the dual‐luciferase reporter assay, treatment with the miR‐130a‐5p analog led to a 40% reduction in the luciferase activity of the wild‐type (WT) 3′UTR reporter, whereas the same treatment led to only a 4% reduction in that of the mutant reporter, indicating the importance of complementary base pairing for miR‐130a‐5p‐induced SPP mRNA degradation (Figure 5D).

Moreover, Schrödinger analysis revealed that elemene docked with the grooves of miR‐130a‐5p through van der Waals forces and pi‐alkyl intermolecular interactions (Figure 5E,F), this binding may affect the function of the miRNA, such as binding to SPP mRNA. Meanwhile, SPP molecular interaction analysis was performed to test the potential for cross‐molecular interactions between miR‐130a‐5p and elemene, and the microscale thermophoresis (MST) trace curve revealed a K_D_ of 505.52 ± 43.039 µm for elemene and miR‐130a‐5p binding, indicating cross‐molecular interaction (Figure 5G; Figure S5G, Supporting Information). Furthermore, to directly assess the regulatory effect of miR‐130a‐5p in a controlled in vitro setting, the synthetic miR‐130a‐5p mimic was exogenously added to the reconstituted RISC complex system. As shown in the bar graph, the addition of miR‐130a‐5p mimic significantly reduced the expression level of SPP mRNA compared to NC. However, co‐treatment with elemene effectively reversed this suppression. When elemene was included, SPP mRNA expression was restored to a level comparable to that of the NC, without a statistically significant difference. These results demonstrate that elemene can directly antagonize miR‐130a‐5p‐mediated degradation of SPP mRNA in a cell‐free RISC complex system (Figure S5H, Supporting Information). These results demonstrated that miR‐130a‐5p induced the degradation of SPP mRNA and that elemene suppressed this process by competitively interacting with miR‐130a‐5p.

To further investigate whether miR‐130a‐5p mediated the regulation of SPP by elemene, HCC cells were treated with elemene in the presence of a miR‐130a‐5p inhibitor or analog. The results showed that the miR‐130a‐5p analog could partially counteract the protective effect of elemene on SPP mRNA, while the miR‐130a‐5p inhibitor reversed the influence of elemene on SPP mRNA degradation (Figure 5H,I; Figure S6A–D, Supporting Information. Therefore, these data indicate that miR‐130a‐5p is the key player mediating the protective effect of elemene on SPP mRNA. Data above demonstrated that elemene induced the SPP/MHC‐I axis by suppressing miR‐130a‐5p‐mediated SPP mRNA degradation in HCC cells.

Growing evidence indicates that miRNAs play crucial regulatory roles in tumor immunotherapy by modulating immune cell function and tumor‐immune interactions.^[^ ^35^, ^36^ ^]^ While the involvement of miRNAs in immune regulation is well‐established, the specific role of miR‐130a‐5p in tumor immunology remains relatively unexplored. The limited research on miR‐130a‐5p in this context highlights the novelty and potential significance of our current investigation.

### 2.6 The miR‐130a‐5p/SPP/MHC‐I Signaling Axis Plays a Key Role in the Immune Microenvironment of HCC

**Figure 6:**
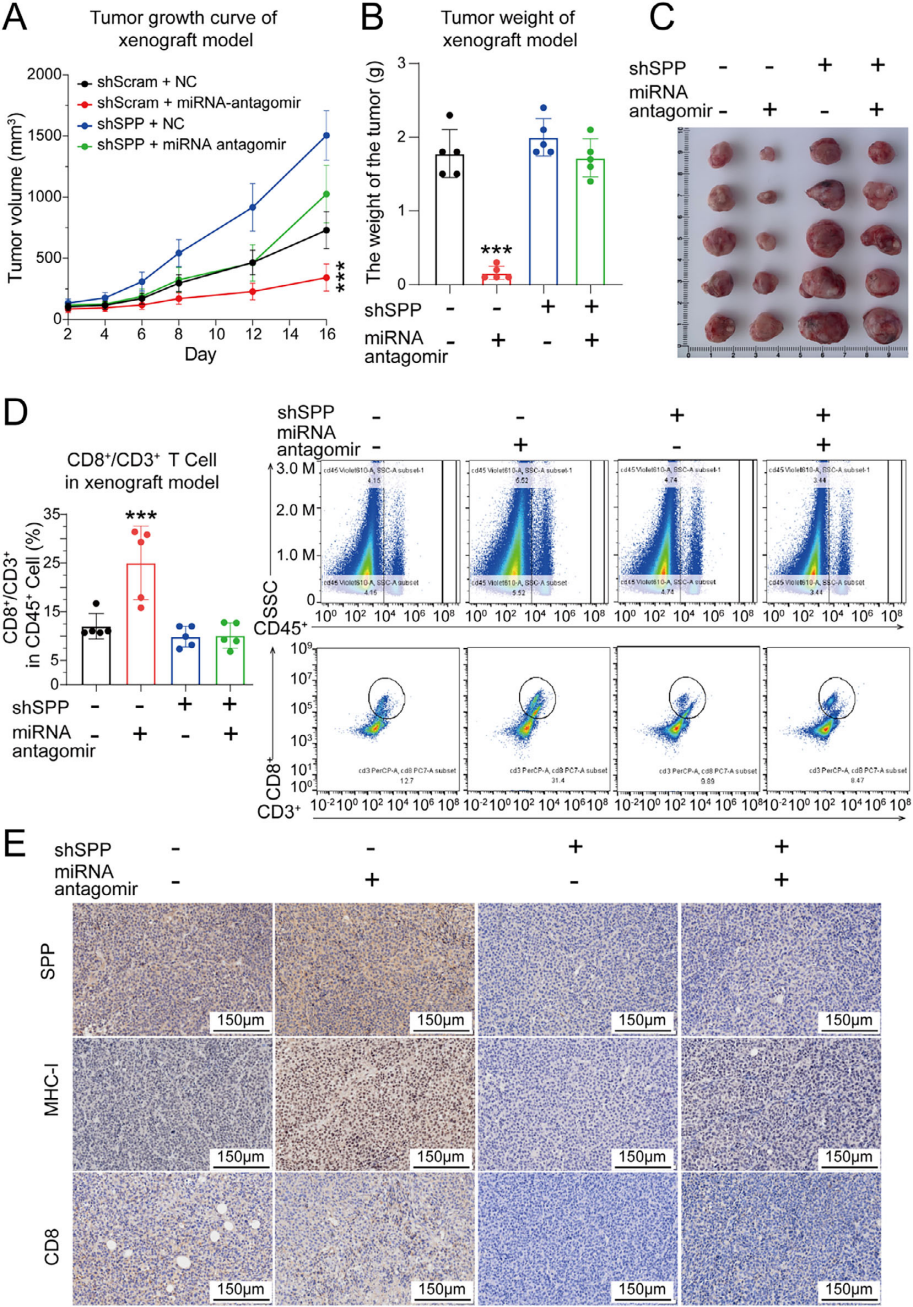
The miR‐130a‐5p antagomir significantly promoted CTL infiltration and inhibited tumor growth via SPP in the subcutaneous xenograft HCC model. A) Tumor growth curves of mice bearing Hepa1‐6‐shscramble and Hepa1‐6‐shSPP xenografts after treatment with the miR‐130a‐5p antagomir. *n* = 5. The data are presented as the means ± SD. (B, C) Histogram of tumor weights B) and photograph of dissected tumors C) from the subcutaneous xenograft HCC model. *n*=5. The data are expressed as the means ± SD. D) Histogram and representative flow cytometry images of CD8^+^/CD3^+^ T cells among CD45^+^ lymphocytes in tumor tissues from the subcutaneous xenograft HCC model after indicated treatment. *n* = 5. The data are presented as the means ± SD. E) Representative IHC images showing the expression of SPP, MHC‐I, and CD8 in tumor tissues from the subcutaneous xenograft HCC model. Scale bar = 150 µm. ^***^ *p* < 0.001.

To further elucidate the role of the miR‐130a‐5p/SPP/MHC‐I axis in TIME regulation, mice bearing Hepa1‐6‐shscramble and Hepa1‐6‐shSPP xenografts were treated with an miR‐130a‐5p antagomir. The tumor volume growth curves revealed that the Hepa1‐6‐shSPP xenografts grew faster than the Hepa1‐6‐shscramble xenografts did, and that intratumoral injection of the miR‐130a‐5p antagomir led to a remarkable decrease in the size of the Hepa1‐6‐shscramble xenograft tumors; however, the same treatment had no effect on the Hepa1‐6‐shSPP xenografts. A similar trend was observed in the weights of the dissected tumors at the end of the experiment (**Figure** **6**A–C). Moreover, immunohistochemical analysis of HCC samples revealed that the miR‐130a‐5p antagomir reduced the signal intensity of Ki‐67 in Hepa1‐6‐shscramble xenografts compared with that in the control group, whereas there was no significant change in Ki‐67 signal intensity in the Hepa1‐6‐shSPP xenografts. The immunohistochemical pattern of cleaved caspase‐3 was opposite that of Ki‐67, which was consistent with the xenograft growth curves (Figure S7A, Supporting Information).

Furthermore, xenograft tissues were subjected to a flow cytometry assay to analyze the proportions of CTLs and CD4^+^ T cells among the total T‐cell population. The results revealed that the miR‐130a‐5p antagomir significantly increased the proportions of CTLs and CD4^+^ T cells among the total T‐cell population in Hepa1‐6‐shscramble xenografts but did not have an obvious influence on Hepa1‐6‐shSPP xenografts (Figure 6D; Figure S7B, Supporting Information). Similarly, the results of the IHC assay revealed that the Hepa1‐6‐shscramble xenografts treated with the miR‐130a‐5p antagomir presented the strongest signal intensities for SPP, MHC‐I, and CD8, which were consistent with the results of the flow cytometry assay. Thus, these findings indicate that the miR‐130a‐5p antagomir reversed the immunosuppressive TIME in a SPP‐dependent manner (Figure 6E). Moreover, the expression levels of miR‐130a‐5p and SPP in the xenograft tissues were measured via qPCR, and the results revealed that the expression of miR‐130a‐5p decreased in all the groups treated with the miR‐130a‐5p antagomir. However, the miR‐130a‐5p antagomir led to an increase in SPP mRNA expression in Hepa1‐6‐shscramble xenografts; this phenomenon was not observed in Hepa1‐6‐shSPP xenografts (Figure S7C,D, Supporting Information).

According to our previous experimental results, increased SPP level enhanced the abundance of MHC‐I/peptide complexes on the surface of HCC cells, so the abundance of MHC‐I/peptide complexes was expected to be reduced by miR‐130a‐5p expression. Therefore, the abundance of membrane MHC‐I was analyzed via flow cytometry in Hepa1‐6‐shscramble and Hepa1‐6‐shSPP cells in the presence or absence of the miR‐130a‐5p inhibitor. The results revealed that the miR‐130a‐5p inhibitor enhanced the MHC‐I fluorescent signal intensity in Hepa1‐6‐shscramble cells, but this enhancement was diminished in Hepa1‐6‐shSPP cells, indicating that miR‐130a‐5p could regulate the abundance of membrane MHC‐I via SPP (Figure S8A,B, Supporting Information). To test the sufficiency of targeting this axis for enhancing MHC‐I abundance, we employed our existing in vivo model using miR‐130a‐5p antagomir alone. The results revealed that the miR‐130a‐5p antagomir enhanced the MHC‐I fluorescent signal intensity in Hepa1‐6‐shscramble tumor, but this enhancement was diminished in Hepa1‐6‐shSPP tumor, indicating that miR‐130a‐5p mainly regulates the abundance of membrane MHC‐I via SPP (Figure S8C,D, Supporting Information). Having established that elemene could induce the SPP/MHC‐I axis by suppressing miR‐130a‐5p, we sought to further determine whether this axis is functionally dependent on the enzymatic activity of SPP as a proteinase. To test this hypothesis, we reconstituted SPP‐knockdown cells with either wild‐type SPP (SPP‐WT) or an enzyme activity‐null mutant (SPP‐D265A) that binds to but cannot cleave its substrates.^[^ ^37^ ^]^ Quantitative analysis (Figure S8E, Supporting Information) and representative flow cytometry histograms (Figure S8F, Supporting Information) of MHC‐I expression revealed that the miR‐130a‐5p inhibitor could not reverse the loss of MHC‐I abundance caused by SPP knocking down, while MHC‐I abundance showed a remarkable increase after the reconstitution of SPP‐WT, but not the catalytically dead SPP‐D265A mutant. This result unequivocally demonstrates that the miR‐130a‐5p/SPP/MHC‐I axis operates in a manner strictly dependent on the cleaving activity of SPP. These data suggested that the miR‐130a‐5p/SPP/MHC‐I axis plays a key role in regulating the TIME of HCC.

Based on all these results, it can be concluded that in parallel to the enhancement of CD8^+^ T cell responses, elemene treatment significantly increased CD4^+^ T helper cell infiltration within TME. This coordinated activation of both T cell subsets suggests that elemene promotes a comprehensive antitumor immune response. CD4^+^ T cells play multifaceted roles in antitumor immunity, including licensing dendritic cells for optimal antigen presentation, sustaining CD8^+^ T cell function and memory formation, and, in certain contexts, directly contributing to tumor cell elimination.^[^ ^38^ ^]^ The expansion of CD4^+^ T cells may potentially stem from enhanced antigen presentation via the SPP/MHC‐I axis, leading to broader T cell activation across both cellular compartments. Alternatively, elemene might independently modulate the tumor microenvironment to favor CD4^+^ T cell recruitment or expansion through additional mechanisms that warrant further investigation. This coordinated enhancement of both CD4^+^ and CD8^+^ T cell responses underscores the potential of elemene to elicit an integrated immune activation strategy that could significantly potentiate the efficacy of immune checkpoint blockade therapy.

### 2.7 SPP is Associated with an Activated Immune Microenvironment in HCC

**Figure 7:**
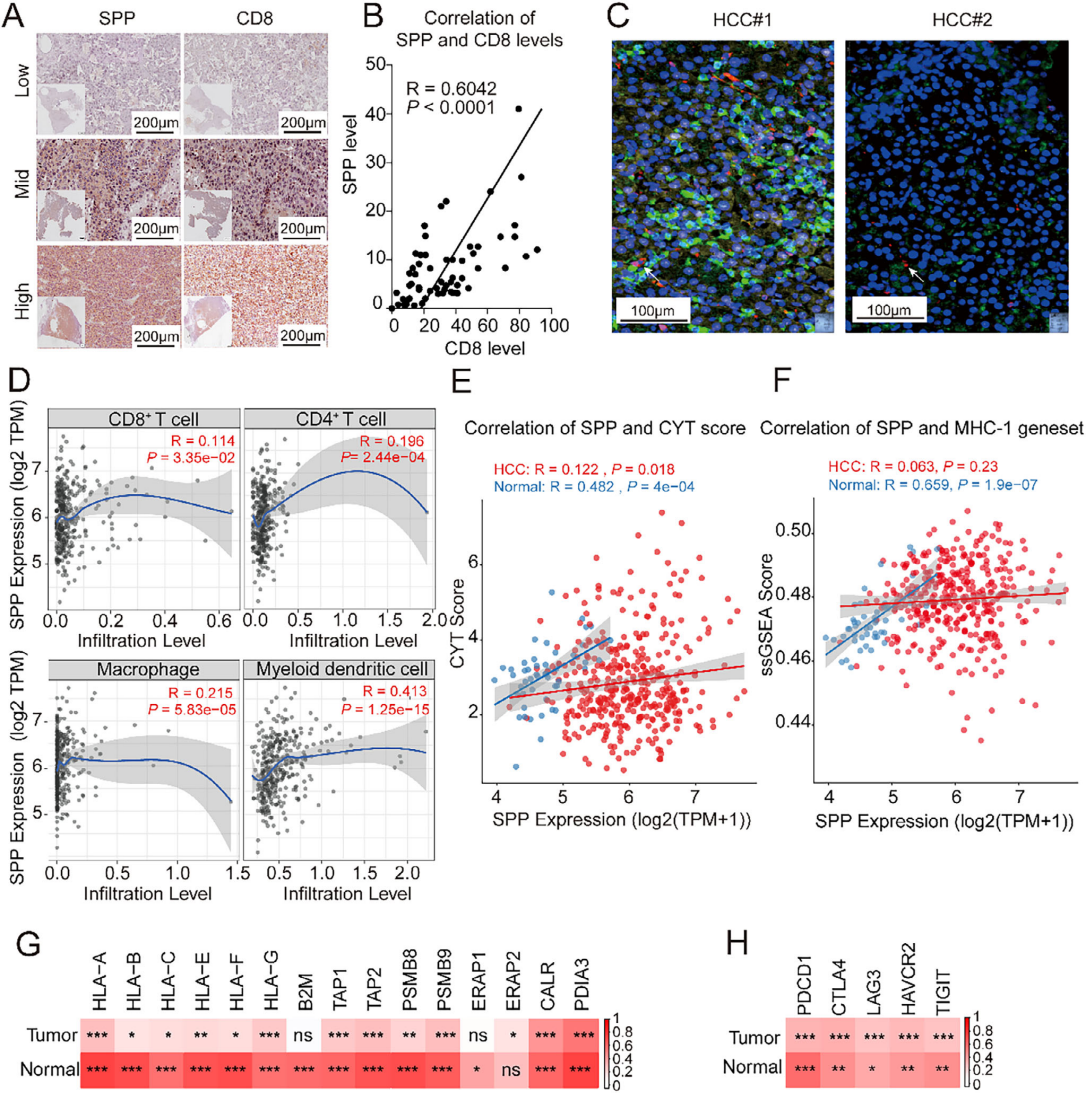
Clinical association of SPP expression with immune cell infiltration and antitumor immunity in HCC. A) Representative immunohistochemical (IHC) images of SPP and CD8 in human HCC tissue samples. Scale bar = 200 µm. B) Scatter plot showing the Pearson correlation between the immunohistochemical signal intensities of SPP and CD8 across a cohort of 60 human HCC samples. C) Representative multiplex immunofluorescence (mIF) images showing the spatial co‐localization of MHC‐I (yellow), SPP (green), and CD8 (red) in HCC samples. Nuclei are counterstained with DAPI (blue). Scale bar = 100 µm. D) Scatter plots showing the correlation between SPP RNA level and the infiltration levels of various immune cells (including CD8^+^ T cells, CD4^+^ T cells, macrophages, and myeloid dendritic cells) in the TCGA‐LIHC database, cohort, as analyzed by the TIMER 3.0 algorithm. E) Scatter plots showing the Pearson correlation analysis between SPP expression level and cytotoxic T lymphocyte activity in both normal and tumor tissues from the TCGA‐LIHC cohort, as measured by the CYT score (calculated from the geometric mean of GZMA and PRF1 gene expression levels). F) Scatter plots showing the correlation between SPP expression and the ssGSEA enrichment scores for the MHC‐I‐mediated antigen processing and presentation pathway in tumor (HCC) and normal (Normal) tissues from the TCGA‐LIHC cohort. G) Heatmap displaying the Pearson correlation coefficients between SPP expression and the mRNA levels of key molecular components of the MHC‐I antigen presentation machinery in the TCGA‐LIHC cohort. H) Heatmap showing Pearson correlation coefficients between the expression levels of SPP and immune checkpoint genes (PDCD1, CTLA4, LAG3, HAVCR2, TIGIT) in the TCGA‐LIHC cohort. ^*^ *p* < 0.05, ^**^ *p* < 0.01, ^***^ *p* < 0.001; ns, nonsignificant.

To translate our mechanistic findings into clinical relevance, we investigated the association between SPP and the tumor immune microenvironment in human HCC cohorts. Analysis of a 60‐sample HCC cohort confirmed a positive correlation between SPP and CD8 protein levels by IHC (**Figure** **7**A,B). Multiplex immunofluorescence further demonstrated spatial co‐localization of SPP, MHC‐I, and CD8^+^ T cells within tumor nests (Figure 7C; Figure S9A, Supporting Information), situating SPP‐expressing cells within an immunologically active context.

Transcriptomic analysis of the TCGA‐LIHC cohort revealed that SPP expression correlated with increased infiltration of multiple immune cell types, including CD8^+^ T and CD4^+^ T cells (Figure 7D). Furthermore, SPP expression was significantly associated with cytotoxic T lymphocyte (CTL) activity, as quantified by the CYT score (geometric mean of GZMA and PRF1 expression). However, the strength of this correlation was markedly attenuated in HCC tumors (R = 0.122, *P* = 0.018) compared to adjacent normal liver tissues (R = 0.482, *p* < 0.001) (Figure 7E), suggesting a dysregulation of the SPP‐CTL functional axis during hepatocarcinogenesis.

A more striking disparity between normal and malignant liver tissues emerged when analyzing the MHC‐I‐mediated antigen presentation pathway. While SPP expression showed a robust positive correlation with the overall pathway activity (ssGSEA score) in normal liver tissues (R = 0.659, *p* < 0.001), this association was virtually abrogated in HCC tumors (R = 0.063, *P* = 0.23) (Figure 7F). Such a pattern of attenuated correlation extended to individual key components of the MHC‐I machinery. As summarized in the heatmap (Figure 7G; Figure S9B, Supporting Information), the strong correlations observed in normal liver tissues across nearly all MHC‐I pathway genes were consistently weakened in HCC tissues. This finding indicates a progressive breakdown of the SPP‐MHC‐I regulatory network during tumorigenesis, potentially reflecting adaptive mechanisms to evade antitumor immunity.

Despite this decoupling from the MHC‐I pathway, SPP‐high tumors retained a distinct immunogenic signature. Specifically, they exhibited significant positive correlations with the expression of multiple immune checkpoint genes, including PDCD1 (PD‐1), CTLA4, and LAG3 (Figure 7H; Figure S9C, Supporting Information). Collectively, this profile defines a classic state of adaptive immune resistance, which is characterized by preserved T cell infiltration, residual cytolytic activity, alongside upregulated inhibitory checkpoints and a disrupted SPP‐MHC‐I antigen presentation axis. In this setting, tumors evade immune‐mediated destruction despite the presence of effector T cells. Importantly, our preclinical data support that elemene therapy can override this resistance by potently upregulating the protein level of SPP, which reconstitutes functional MHC‐I‐mediated antigen presentation and thereby creates a therapeutic vulnerability that can be exploited by checkpoint blockade. These clinical observations reinforce the translational potential of targeting the SPP axis to sensitize HCC to immunotherapy.

## 3 Discussion

In this study, we discovered that combination therapy with elemene and anti‐PD‐1 had synergistic anti‐HCC effects and elucidated the role of the miR‐130a‐5p/SPP/MHC‐I axis in the elemene‐induced reactivation of the TIME. Moreover, preliminary toxicity analysis indicated a good safety profile for this combination of two medicines may be widely applied in clinical practice, suggesting high potential for further clinical translation.

Immunotherapies have changed the landscape of cancer treatment during the past 2 decades, especially ICIs, which target PD‐1/PD‐L1.^[^ ^39^ ^]^ However, reports of immune‐related adverse events (irAEs), including dermatological, gastrointestinal, and renal toxicities associated with ipilimumab; arthralgia, pneumonitis, and hepatotoxicity associated with pembrolizumab; endocrine toxicity associated with nivolumab; and hypothyroidism associated with atezolizumab, have emerged as drug consumption has steadily increased.^[^ ^40^ ^]^ Therefore, many research groups are working on tumor‐targeted delivery strategies mediated by nanotechnology techniques to reduce the systemic toxicity of anti‐PD‐1/PD‐L1 antibodies.^[^ ^41^, ^42^ ^]^ As a terpenoid with a small molecular weight (204.35), pure elemene is a volatile liquid, but it can be encapsulated in liposomes to overcome this problem during clinical formulation. Elemene‐liposomes are commonly used as a monotherapy or in combination with radiotherapy and chemotherapy for the management of malignant tumors. As demonstrated by numerous in vitro and in vivo experiments, liposomes tend to become passively enriched in solid tumors (especially HCC tumors) via the blood circulation due to the abnormal hydrodynamic characteristics of the intratumoral vasculature. Since our study demonstrated that elemene and anti‐PD‐1 exhibit synergistic anti‐HCC effects when they are administered separately, we speculate that a composite elemene‐liposome with anti‐PD‐1 surface decoration might have more potent synergetic efficacy. Soon, further collaborative research with nanomedicine experts should be conducted in parallel with our clinical evaluation of elemene and anti‐PD‐1 combination therapy.

CTLs are a current focus of cancer immunotherapy, but recent studies have revealed that more types of cells, such as NK cells and macrophages, are also promising targets for cancer immunotherapy.^[^ ^43^, ^44^, ^45^ ^]^ Our study elucidates a multilayered immunomodulatory mechanism of elemene mediated through the miR‐130a‐5p/SPP axis. We demonstrate that in tumors with intact SPP expression, miR‐130a‐5p antagomir treatment significantly enhanced tumor infiltration of CD8^+^T cells, CD62L^+^ central memory T cells, NK1.1^+^ natural killer cells, and CD11c^+^ dendritic cells, effects that were completely abolished in SPP‐knockdown tumors (Figures S10A,B and S11A,B Supporting Information), establishing SPP's indispensable role in this pathway. Notably, the unchanged PD‐1 and MHC‐II levels suggest a mechanistically specific enhancement of immune recognition through SPP‐mediated MHC‐I antigen presentation without exacerbating T‐cell exhaustion (Figures S10C and S11C, Supporting Information). These findings align with and extend previous reports of elemene's dual immunomodulatory capabilities, while our current work establishes its role in enhancing tumor immunogenicity via MHC‐I upregulation, earlier studies have documented its ability to reprogram macrophage polarization from M2 to M1 phenotype ^[^ ^46^ ^]^ and disrupt tumor‐promoting crosstalk between cancer cells and M2 macrophages.^[^ ^47^ ^]^ Together, these insights not only establish the crucial position of the miR‐130a‐5p/SPP/MHC‐I axis in elemene's action but also provide a novel theoretical framework for cancer immunotherapy based on multi‐target coordination, highlighting how molecular pharmacology approaches can reveal complex immune network regulation.

The proteinase SPP was identified as a key mediator of the immune‐activating functions of elemene in our research, and the loss of SPP was associated with a suppressive TIME resulting from fewer membrane peptide/MHC‐I complexes in HCC cells. In addition to HCC, our preliminary study on patient‐derived organoids also revealed that elemene‐induced SPP expression might affect peptide/MHC‐I complexes in other types of solid tumors in a similar manner. However, some studies have shown that SPP may play either a tumor suppressive or oncogenic role depending on the type of cancer. For example, Aurélie Durgeau et al. studied LC specimens and reported that high SPP expression was a positive indicator for immunotherapy.^[^ ^48^ ^]^ Yang H et al. and Wei JW et al. reported that SPP (named HM13 in their reports) has oncogenic functions in BC and glioblastoma.^[^ ^49^, ^50^ ^]^ While our data establish the miR‐130a‐5p/SPP/MHC‐I axis as a critical mechanism, we cannot rule out that other elemene‐induced changes contribute to its overall immunostimulatory and antitumor effects, potentially in an additive or synergistic manner with enhanced antigen presentation. Therefore, even though elemene exhibits antiproliferative effects in many types of cancer cells, our discovery regarding the synergy between elemene and anti‐PD‐1 for HCC immunotherapy should not be directly applied to other types of cancer, and the biological mechanism underlying the drug‐drug interaction might not be confined to the miR‐130a‐5p/SPP/MHC‐I axis. Future work will be needed to compare the peptide repertoire at the amino acid sequence level in the presence or absence of SPP expression in a wider range of HCC cells, which would directly clarify its specific contribution to the MHC‐I loading process in HCC.

Our analysis of human HCC cohorts reveals a critical pathophysiological insight: the SPP‐high tumor microenvironment exists in a state of functional decoupling. While it is spatially structured for immune engagement and broadly inflamed, the crucial functional links between SPP and both CTL cytotoxicity and the MHC‐I antigen presentation pathway are specifically weakened in tumors compared to the normal liver. This suggests that HCC cells have undergone functional adaptations to disrupt the very effector functions that SPP is meant to orchestrate. In this context, elemene acts to reconstitute this disabled SPP‐MHC‐I functional axis, thereby converting a state of dysfunctional immune recognition into one of productive immune alert and creating the vulnerability to checkpoint blockade that we observe.

Our elucidation of the miR‐130a‐5p/SPP/MHC‐I axis not only provides mechanistic insights into elemene‐induced immunogenicity but also unveils actionable therapeutic strategies for HCC. From a pathophysiological perspective, downregulation of MHC‐I is a well‐documented immune evasion mechanism in human HCC, often correlated with poor response to ICI.^[^ ^51^, ^52^ ^]^ Our findings suggest that targeting this axis using miR‐130a‐5p antagonists could restore MHC‐I‐mediated antigen presentation and convert immune‐cold tumors into immune‐hot phenotypes, thereby expanding the subset of patients eligible for ICIs therapy. Notably, the precision of this mechanism stands in contrast to global immunomodulatory approaches, such as gut microbiome‐based therapies. While fecal microbiota transplantation or probiotic interventions have shown promise in enhancing ICIs efficacy,^[^ ^53^, ^54^ ^]^ their mechanisms remain pleiotropic and poorly defined, often involving ecosystem‐wide changes in microbial metabolites, barrier integrity, and innate immunity.^[^ ^55^ ^]^ In comparison, the miR‐130a‐5p/SPP/MHC‐I axis offers a druggable target with measurable biomarkers for patient stratification and response monitoring. This molecular definition could facilitate the development of combination therapies wherein microbiome modulation primes the host immune system broadly, while axis‐targeted agents precisely restore tumor‐specific antigen presentation. Future clinical trials should validate whether targeting this axis, particularly in patients with low SPP/high miR‐130a‐5p expression, can synergize with ICB and/or microbiome‐based therapies to overcome resistance in HCC.

Overall, we found that the elemene augments the effects of anti‐PD‐1 immunotherapy on HCC through regulating the miR‐130a‐5p/SPP/MHC‐I axis. In detail, elemene reactivates the TIME by promoting antigen presentation, thereby exhibiting synergistic anti‐HCC effects with anti‐PD‐1. Thus, combining elemene and anti‐PD‐1 therapy may be a safe and effective strategy for HCC treatment with high translational potential. Our discovery regarding the synergy between elemene and anti‐PD‐1 may inspire further mechanistic studies on clinically validated combinations of traditional Chinese and Western medicines. While our preclinical data strongly support the combination of elemene with anti‐PD‐1 therapy, direct clinical validation is the essential next step. As an immediate and translational extension of this work, we propose a prospective pilot study monitoring dynamic changes in plasma miR‐130a‐5p and PBMC transcriptomes in patients receiving this combination. This liquid biopsy approach provides a minimally invasive strategy to validate our mechanistic axis and identify potential predictive biomarkers for a larger, biomarker‐enriched randomized controlled trial in the future.

## 4 Experimental Section

### Cell Culture

The Hepa1‐6 cell line (RRID: CVCL_0327) was provided by the Hospital of Zhejiang University, sourced from the China Center for Type Culture Collection. The Hepa1c1c7 cell line (RRID: CVCL_0328) was gifted by Prof. Shimei Zhuang from Sun Yat‐Sen University, sourced from American Type Culture Collection (ATCC). Huh1 (RRID: CVCL_2956) and HEK‐293T (RRID: CVCL_0063) were obtained from ATCC. These cells were cultured in high‐glucose Dulbecco's modified Eagle medium (DMEM) supplemented with 10% heat‐inactivated fetal bovine serum (FBS) and 1% penicillin/streptomycin at 37 °C with 5% CO_2_ in a humidified incubator. The cells were passaged for ≤ 3 months from the frozen early passage stocks that had been received from the indicated sources. During the study, all the cell cultures were periodically tested for mycoplasma using MycoAlert Mycoplasma detection kits (Lonza, #LT07‐318).

### In vivo Treatment of Murine HCC Models

All animal experiments were approved by the Animal Experiment Ethical Inspection Committee of Hangzhou Normal University (reference number: HSD‐20241122‐03). Male C57BL/6J mice (all aged 6–8 weeks) were obtained from GemPharmatech (Nanjing, China).

To construct the HCC xenograft model, 0.1 mL of cell suspension was injected subcutaneously into the left flank of each mouse (≈1.0 × 10^5^ cells per mouse). The volumes of the tumors were determined every 2 days using calipers, and the mice were sacrificed humanely once the tumor volume reached 1500 mm^3^. Subcutaneous tumor volume was calculated using the following equation: V = (A × B^2^)/2, where V represents the volume of the tumor; A represents the maximum diameter of the tumor; and B represents the minimum diameter of the tumor.

To construct the orthotopic HCC model, luciferase‐labeled Hepa1‐6 cells were resuspended at a density of 1.0 × 10^5^ cells per mL, and the cell suspension was mixed with an equal volume of Matrigel matrix. C57BL/6J mice were anesthetized via an intraperitoneal injection of 0.3% pentobarbital (0.1 mL/10 g). An incision was then made ≈1 cm under the xiphoid of each mouse using scissors, and the left lobe of the liver was gently squeezed out. Next, 10 µL of the cell mixture was injected into the left lobe of the liver (≈1.0 × 10^5^ cells per mouse). After the Matrigel mixture had set, the left lobe was placed back into the abdomen, and the abdominal incision was sutured. D‐Luciferin potassium salt was injected intraperitoneally to visualize the location and size of each tumor 1 week after orthotopic implantation, and the signals were imaged using the IVIS Spectrum In Vivo Imaging System (PerkinElmer, USA). Imaging was performed twice a week, and the mice were sacrificed at the end of the fifth imaging cycle.

EP therapy in C57BL/6J mice: Treatment started on the 5th day after tumor cell inoculation. Elemene was administered via tail vein injection every other day at a dosage of 50 mg kg^−1^, and anti‐PD‐1 was given twice a week via tail vein injection at a dosage of 100 µg mouse^−1^ (five injections in total). The tumor‐bearing mice were euthanized on day 17 after the start of treatment. Tumor tissues were analyzed via immunohistochemical staining, and TILs were analyzed via flow cytometry.

miR‐130a‐5p antagomir therapy: Mice were intratumorally injected with the control or antagomir on the 5th day after tumor cell inoculation, and tumor sizes were measured every 2 days with calipers. The tumor‐bearing mice were euthanized on day 17 after the start of treatment. TILs were analyzed via flow cytometry.

For survival analysis, an orthotopic HCC model was established as described above. The number of dead mice was recorded daily, and the remaining mice were euthanized on day 24 after the start of EP treatment. Statistical analysis was performed using GraphPad Prism 8 software. Kaplan‐Meier survival curves and the corresponding log‐rank (Mantel‐Cox) tests were used to evaluate the significant differences between groups in the survival studies. A value of P < 0.05 was considered to indicate statistical significance.

Survival analysis was performed with *n* = 10 mice per group to ensure sufficient statistical power for long‐term outcome studies. Antitumor efficacy studies and flow cytometry analyses used *n* = 3–6 mice per group, as these assays yield highly consistent and quantifiable readouts within established experimental models, and the sample sizes were consistent with common practices in the field for such endpoints.

### Cytokine Array of Peripheral Blood

On the 17th day of EP treatment, blood was collected from the tumor‐bearing mice and incubated statically for 1 h at room temperature. Then, the serum was isolated by centrifugation at 1000 rpm for 30 min to remove the clot.

The LEGENDplexTM assay (BioLegend, #740134) was used to evaluate the serum concentrations of multiple cytokines. Beads of a specific size and internal fluorescence intensity were conjugated to the capture antibody against a cytokine of interest. Then, beads for the selected cytokines were mixed and incubated with serum. After washing the beads, a biotinylated detection antibody cocktail against the same set of cytokines was added to form capture bead–analyte–detection antibody sandwiches. Streptavidin–phycoerythrin (SA‐PE) was subsequently added, and the beads were subjected to flow cytometry analysis. Bead identity was determined according to bead size and internal fluorescence intensity, and cytokine quantification was performed according to the fluorescence intensity of PE. The concentration of a particular analyte was determined using the standard curve provided by the corresponding assay kit.

### Isolation and Flow Cytometric Analysis of Tumor‐Infiltrating Immune Cells

Tumor tissue was isolated from the mice and cut into pieces for milling, and a cell suspension was prepared by repeatedly rinsing with DMEM. The cell suspension was filtered through a 70‐µm filter to form a single‐cell suspension. The lymphocytes were isolated via density gradient centrifugation using 70% OptiPrep (Sigma, #D1556). Then, the tumor‐infiltrating lymphocytes (TILs) were collected, resuspended in FcR blocking reagent for 10  min, and then stained with a dead cell indicator and fluorochrome‐conjugated antibodies targeting surface markers in staining buffer (BioLegend, #420201) for 30 min on ice. Then, the cells were washed with wash buffer (BioLegend, #421002) and analyzed via flow cytometry (BD Biosciences, USA) following the exemplified gating strategy for flow cytometry analysis. The data were processed using FlowJo V10 software.

### TCR‐Seq

RNA samples were analyzed via high‐throughput sequencing of TRA using the deep ImmuHub TCR profiling system by ImmuQuad Biotech (Hangzhou, China). Briefly, a 5′‐RACE unbiased amplification protocol was used, which incorporates unique molecular barcodes (UMBs) introduced during cDNA synthesis to control bottlenecks and eliminate errors from PCR and sequencing. Sequencing was performed on an Illumina NovaSeq system in PE150 mode (Illumina, USA). One common adaptor with a UMB was added to the 5′ end of the cDNA during first‐strand cDNA synthesis, and one reverse primer corresponding to the constant (C) regions of each TRA was designed to facilitate PCR amplification of the cDNA sequences in a less biased manner. The UMB attached to each raw sequence read was applied to correct the PCR and sequencing errors, and the PCR duplicates were removed. The V, D, J, and C segments were mapped with NCBI, the CDR3 regions were extracted, and the clonotypes for all the clones were assembled. The resulting nucleotide and amino acid sequences of the CDR3 of TRA were determined, and those with out‐of‐frame and stop codon sequences were removed from the identified TRA repertoire. The number of each TRA clonotype was determined by adding the number of TRA clones sharing the same nucleotide sequence as CDR3.

### Immunoprecipitation/Mass Spectrometry (IP/MS)

The cells were lysed on ice using IP lysis buffer (20 mm Tris (pH 8.0), 10% glycerol, 150 mm NaCl, 0.1% NP‐40, 0.1 mm EDTA). Protein lysates were precleaned with protein A/G magnetic beads (Thermo Fisher, #88802) and incubated overnight at 4 °C with the indicated antibodies or with an isotype control IgG. The immunocomplexes were collected with a magnetic rack, washed with lysis buffer, and then stored in 2× SDS sample loading buffer. The eluents were subjected to liquid chromatography/mass spectrometry (LC/MS) sequencing and data analysis by Oebiotech Co., Ltd. (Shanghai, China).

### MST

MST was performed by Bio‐Lab Technology Co., Ltd. (Wuhan, China). During the experiment, the 5′ end of miR‐130a‐5p was labeled with a Cy5 tag and called the target, and elemene was used as the ligand. The ligand solution was subsequently diluted with a 2‐fold concentration gradient to generate a total of 16 concentrations, and these solutions were placed in tubes and mixed equally with the target. After being drawn into the capillary, each mixture was placed in a Monolith NT.115 MST instrument with a temperature gradient, and the molecules moved from the high‐temperature area to the low‐temperature area. The excitation light induced the target to emit fluorescence, and the change in fluorescence intensity of the emitted light was detected to record the difference in thermal drift speed.

### Cell Isolation and Organoid Culture

Human fresh tissue samples obtained from patients with CC, HCC, ICC, or LC by surgical resection were divided into four parts, which were used for DNA extraction, RNA extraction, histopathological identification, and organoid culture. Organoid culture was performed as previously described.^[^ ^56^, ^57^ ^]^ The tissues intended for organoid culture were washed three times with PBS containing 1% penicillin/streptomycin, then placed in an EP tube, minced, and digested with 1.5 mg mL^−1^ type 1 Collagenase (Worthington, #LS004197) containing 1% penicillin/streptomycin for 1 to 2 h at 37 °C until a large number of cell masses formed. The digested suspension was filtered through a 100 µm cell strainer and centrifuged at 200–300 g for 5 min. After discarding the supernatant, the pellet was washed with Advanced DMEM/F12 (Gibco, #12634‐010), centrifuged again, and mixed with Matrigel (Corning, #356231). 2000–5000 cells were seeded per well in 24‐well plates. After allowing for coagulation, organoids were cultured using specific media: CRC organoid medium (OrganPharma, #NGH02001), HCC organoid medium (OrganPharma, #NGH02004), ICC organoid medium (OrganPharma, #NGH02005), and LC organoid medium (OrganPharma, #NGH020011).

Histological staining of organoids was carried out as follows. Organoids, which were treated with elemene for 48h, were collected from the culture plates and fixed with 4% paraformaldehyde for 30 min. After centrifugation, the supernatant was discarded, and the organoids were washed with PBS 3 times. Finally, the organoid pellet was mixed with LM Agarose (Coolaber, #CA1351‐5g), allowed to solidify, followed by dehydration with a gradient of ethanol, xylene clearing, and embedding in paraffin. The samples were then sectioned into 5 µm slices. Immunohistochemical staining was performed using SPP and MHC‐I antibodies to evaluate the expression of SPP and MHC‐I in organoids derived from different tumors following treatment with elemene.

### Molecular Docking of Elemene with miR‐130a‐5p

The structure of the miR‐130a‐5p was predicted using the AlphaFold server (https://alphafoldserver.com/), and the structural model with the highest Predicted Local Distance Difference Test(pLDDT) score was selected as the final structure for subsequent analysis. The structure of elemene was obtained from PubChem (https://pubchem.ncbi.nlm.nih.gov/). The docking simulation was processed by Schrödinger Glide v2021.3 software, and the result was visualized through Discovery Studio 2021 Client and PyMOL v2.6.

### Statistical Analysis

Statistical analysis was performed using the GraphPad Prism 8 software. The quantified data were presented as the means ± SD or means ± SEM from three independent experiments unless otherwise stated. Continuous data were compared using unpaired Student's t tests (comparisons between two variables) or one‐way ANOVA with the Bonferroni post hoc correction (comparisons of multiple variables). Survival curves were generated according to the Kaplan–Meier method. A value of *p* < 0.05 was considered to indicate statistical significance.

## Conflict of Interest

The authors declare no conflict of interest.

## Author Contributions

M.W., M.S., and H.D. contributed equally to this work. M.W., J.C., Y.Q., and T.X. contributed to conceptualization. M.W., M.S., H.D., and X.Z. performed formal analysis. M.W., M.S., H.D., X.Z., J.Z., Z.Z., and M.N. carried out the investigation. X.Z., Z.Z., M.N., L.L., X.Y.C., J.H., Q.L., F.B., and X.L.C. developed the methodology. H.D., J.C., Y.Q., and T.X. provided resources. M.S., Y.P., and L.J. handled software. X.Z. and J.Z. performed validation. H.D., M.W., J.C., Y.Q., and T.X. acquired funding. M.W., J.C., Y.Q., and T.X. managed project administration. J.C., Y.Q., and T.X. supervised the work. M.W., M.S., H.D., and Y.Q. wrote the original draft; and M.W., M.S., H.D., Z.F., J.C., Y.Q., and T.X. reviewed and edited the manuscript.

## Supporting information



Supporting Information

## Data Availability

The data that support the findings of this study are available in the supplementary material of this article.
