# Supplementary material for: Elemene Augments the Effects of Anti‐PD‐1 Immunotherapy on Hepatocellular Carcinoma by Regulating the miR‐130a‐5p/SPP/MHC‐I Axis
Source: Adv Sci (Weinh). 2026 Jan 12;13(11):e11887. doi: 10.1002/advs.202511887 (PMC12931222; doi:10.1002/advs.202511887)
Supplement: Supplementary file 1 — Supporting Information [file ADVS-13-e11887-s001.docx]

**Supporting Information**

**Elemene Augments the Effects of Anti-PD-1 Immunotherapy on Hepatocellular Carcinoma by Regulating the miR-130a-5p/SPP/MHC-I Axis**

Menglan Wang^1†^, Mengqing Sun^1†^, Heng Dong^1†^, Xiaoting Zhang^1^, Jingbo Zhang^1^, Zhengguo Zhang^1^, Mengjie Ni^1^, Lina Li^1, 3^, Yunxin Pei^1^, Xiaoyu Chen^1^, Qian Li^1^, Fangtian Bu^1^, Jiacheng Huang^2^, Liangyu Jiang^1^, Zhuting Fang^4^, Xuliang Chen^5^, Jianxiang Chen^1*^, Yiting Qiao^2*^, Tian Xie^1*^

**Materials and Methods**

**Drugs and antibodies**

The following agents and antibodies used in this study were obtained from the indicated companies: Elemene Injectable Emulsion (Dalian Huali Golden Port Pharmaceutical Co. Ltd., National Medicine, approval number: H10960115); InVivoMab anti-mouse-PD-1 (BioXCell, #BE0146); α-SPP antibody (Lifespan, #LS-B15247-50); α-GAPDH antibody (Proteintech, #60004-1); α-mouse-H-2K^b^ MHC class I antibody (BioLegend, #AF6-88.5); α-CD8 antibody (Santa Cruz, #sc-7970); α-IFN-γ antibody (Invitrogen, #MM701); α-Granzyme B antibody (Abcam, #ab255598); the Fixable Viability Kit (BioLegend, #423106); α-mouse CD45-Brilliant Violet 605^TM^ antibody (BioLegend, #103140); α-mouse CD3ε-PerCP/Cyanine 5.5 antibody (BioLegend, #100328); α-mouse CD4-KIRAVIA Blue 520^TM^ antibody (BioLegend, #100478); α-mouse CD8a-PE/Cyanine 7 antibody (BioLegend, #100722).

**Plasmids and lentiviral infection**

To establish stably silenced SPP cell lines, the shSPP oligomers were designed, annealed, and cloned into lentiviral cloning vectors pLKO.1 using EcoRI and AgeI cloning sites and verified by Sanger sequencing. shSPP-1 (5’-3’: CACCATCTTCATCATGCACAT), shSPP-2 (5’-3’: GAGATCATCAACTATGAGTTT), shScramble (5’-3’: CCTAAGGTTAAGTCGCC). Lentiviral were produced in 293T cells by co-transfecting with the pLKO.1 vectors, an envelope plasmid and two packing plasmids, and infected Hepa1-6 and Hepa1c1c7 cells in the presence of 5 μg/mL polybrene (Santa Cruz, #sc-134220). Stably transfected cells were then screened using puromycin (Beyotime, #ST551). Knockdown efficiency was assessed by qPCR and WB analysis. The wild-type human SPP cDNA and its catalytic inactive mutant (SPP-D265A) were synthesized and cloned into the pLVX-IRES-puro vector by Tsingke Biotechnology Co., Ltd. The integrity of all constructs was confirmed by DNA sequencing.

**Western blot**

The cells and tissues were lysed with ice-cold RIPA lysis buffer (Thermo Fisher, #89901) containing a protease inhibitor cocktail (Roche, #04693132001) to extract total proteins, and the protein concentration was determined using a BCA protein assay kit (Thermo Fisher, #23225). Then, equal amounts of protein samples were loaded on SDS-PAGE gels for separation and then transferred to a PVDF membrane, which was blocked in 5% skim milk. After sequential incubation with the primary and secondary antibodies, the blots were detected using a Western Bright Sirius kit (Advansta, #K-12043) and a ChemiDoc Imaging System (Bio-Rad, USA).

**Serum indices of liver function**

To assess liver function, the serum levels of alanine aminotransferase (ALT), albumin (ALB), globulin (GLB), total protein (TP) and aspartate aminotransferase (AST) were determined via a microplate method with an automatic biochemical analyzer (HITACHI, Japan). These analyses were carried out by the Animal Experiment Center of Hangzhou Normal University. All procedures were conducted in accordance with the instructions.

**Histological and immunohistochemical assays**

Sixty samples were obtained from HCC patients who underwent hepatectomy at The First Afﬁliated Hospital, Zhejiang University School of Medicine. This study was approved by the Clinical Research Ethics Committee of the First Affiliated Hospital, Zhejiang University School of Medicine (reference number: IIT20200432A).

Both the clinical tumor samples and the tumor tissues from the animal models were fixed in formalin, embedded in paraffin, sectioned and subjected to hematoxylin and eosin (H&E) staining. After deparaffinization, the following steps were performed in sequence for immunohistochemical analysis: antigen retrieval, endogenous peroxidase blocking, serum blocking, overnight incubation with the primary antibody, incubation with the secondary antibody, DAB chromogenesis (Proteintech, #PR30010), restaining the nuclei, dehydration and sealing. The prepared sections were observed and photographed with a fully automated slide scanning imaging system (OLYMPUS, Japan).

**Real-time PCR and RNA-Seq**

Total RNA was extracted using Total RNA Extraction Reagent (Vazyme, #R401-01-AA), and cDNAs were reverse-transcribed using HiScript II Q Select RT SuperMix (Vazyme, #R323-01) or the miRNA 1st Strand cDNA Synthesis Kit (by stem-loop) (Vazyme, #MR101-01) for miRNA following the manufacturer’s guidelines. Quantitative real-time PCR (qRT-PCR) of the mRNAs and miRNAs in this study was performed using the ChamQ Universal SYBR qPCR Kit (Vazyme, #Q711) on a CFX Connect Real-Time PCR Detection System (Bio-Rad, USA). The primer sequences are listed in table S3.

RNA sequencing was performed by LC Sciences (Hangzhou, China). In brief, RNA was isolated with TRIzol (Thermo Fisher, #15596018) and quantified with a NanoDrop ND-1000 (Thermo Fisher, USA). RNA integrity was detected with a Bioanalyzer 2100 (Agilent, USA). Oligo(dT) magnetic beads (Thermo Fisher, #25-61005) were used to capture mRNA containing PolyA through two rounds of purification. The captured mRNA was fragmented using the NEBNext RNA Fragmentation Module (New England Biolabs, #E6150S). cDNA was synthesized from the fragmented RNA using Invitrogen SuperScriptTM II Reverse Transcriptase (Invitrogen, #1896649). Then, *Escherichia coli* DNA polymerase I (New England Biolabs, #m0209) and RNase H (New England Biolabs, #m0297) were used for double-strand DNA synthesis. dUTP solution (Thermo Fisher, #R0133) was added to ensure specificity, followed by the addition of an A base to each end. The two chains were digested with the enzyme UDG (New England Biolabs, #m0280), kept at 95°C for 3 min, denatured at 98°C for a total of 8 cycles (15 sec each), annealed at 60°C for 15 sec, extended at 72°C for 30 sec, and finally extended at 72°C for 5 min to form a 300 bp ± 50 bp fragment size library. The library was subjected to double-end sequencing using an Illumina NovaSeq^TM^ 6000 in PE150 mode according to standard procedures.

miRNA sequencing was performed by LC Sciences (Hangzhou, China). In brief, the miRNA sequencing library was constructed using the TruSeq Small RNA Sample Preparation Kit (Illumina, #RS-200-0036) according to the manufacturer's instructions and sequenced on the Illumina HiSeq 2000/2500 platform, which allows for 3-5 million 50 bp single‐end reads. After quality control of the raw sequencing data, the data were mapped against the Rfam and Repbase databases to annotate the miRNAs. Finally, normalization was used to identify the differentially expressed miRNAs.

**RNA decay analysis**

The cells were pretreated with elemene or the miR-130a-5p mimic or inhibitor for 24 h. Then, the cells were treated with actinomycin D (MCE, #HY-17559) at various time points (0, 2, 4, 8, 10, 12, and 18 h) prior to harvesting. Total RNA was subsequently extracted, and the expression levels of SPP mRNA were quantified via qRT-PCR. The SPP level at each time point was compared to that at 0 h to calculate the percent mRNA remaining, after which the decay rate of SPP mRNA was quantified via nonlinear regression curve fitting (one-phase exponential decay) using GraphPad Prism 8 software.

**Luciferase reporter assay**

The 3’UTR sequence of SPP mRNA, including the putative miR-130a-5p binding site (WT) or a mutant form of this binding site (mutant, MUT), was synthesized by Tsingke Biotechnology (Beijing, China), cloned and inserted into the pmirGLO vector. This construct was then cotransfected into Hepa1-6 cells along with miR-130a-5p mimics or a negative control (NC). Luciferase activity was subsequently measured using a Dual Luciferase Reporter Assay Kit (Vazyme, #DL101-01).

**mRNA targeted degradation mediated by AGO2 overexpression**

The 3'-untranslated region (3'UTR) sequence of SPP mRNA, containing the predicted miR-130a-5p binding site, was synthesized by Tsingke Biotechnology Co., Ltd. (Beijing, China) and subsequently cloned into the pmirGLO vector. The constructed plasmid was transfected into HEK293T cells, and total RNA was extracted 24 hours post-transfection.A human AGO2 gene expression plasmid with HA tag was synthesized by Hangzhou Rapid Biological Technology Co., Ltd. HEK293T cells were first transfected to overexpress HA-tagged Ago protein. Cytoplasmic lysates with biological activity were then prepared using mild lysis buffer. The lysates were mixed with miR-130a-5p and incubated at 30°C for 1 hour to form the RISC complex system. The total RNA containing overexpressed SPP mRNA 3'UTR was added to the RISC complex system, along with different concentrations of elemene. After incubation at 37°C for 0 or 1 hour, total RNA was extracted and the expression level of SPP mRNA was quantified by qRT-PCR using GAPDH as internal control.

**Bioinformatic analysis of public cohorts**

Transcriptomic and clinical data for the TCGA-LIHC cohort (374 tumors, 50 normals) were analyzed. The infiltration levels of various immune cells were estimated using the TIMER 3.0 algorithm. Cytolytic activity (CYT score) was calculated as the geometric mean of GZMA and PRF1expression. The activity of the MHC-I antigen presentation pathway was quantified via single-sample GSEA (ssGSEA) using the KEGG gene set. Pearson correlation analysis was employed to assess the relationships between SPP expression and immune cell infiltration, CYT score, pathway activity, and the expression of MHC-I-related and immune checkpoint genes. *P* < 0.05 were considered significant.

**Supplementary figures**


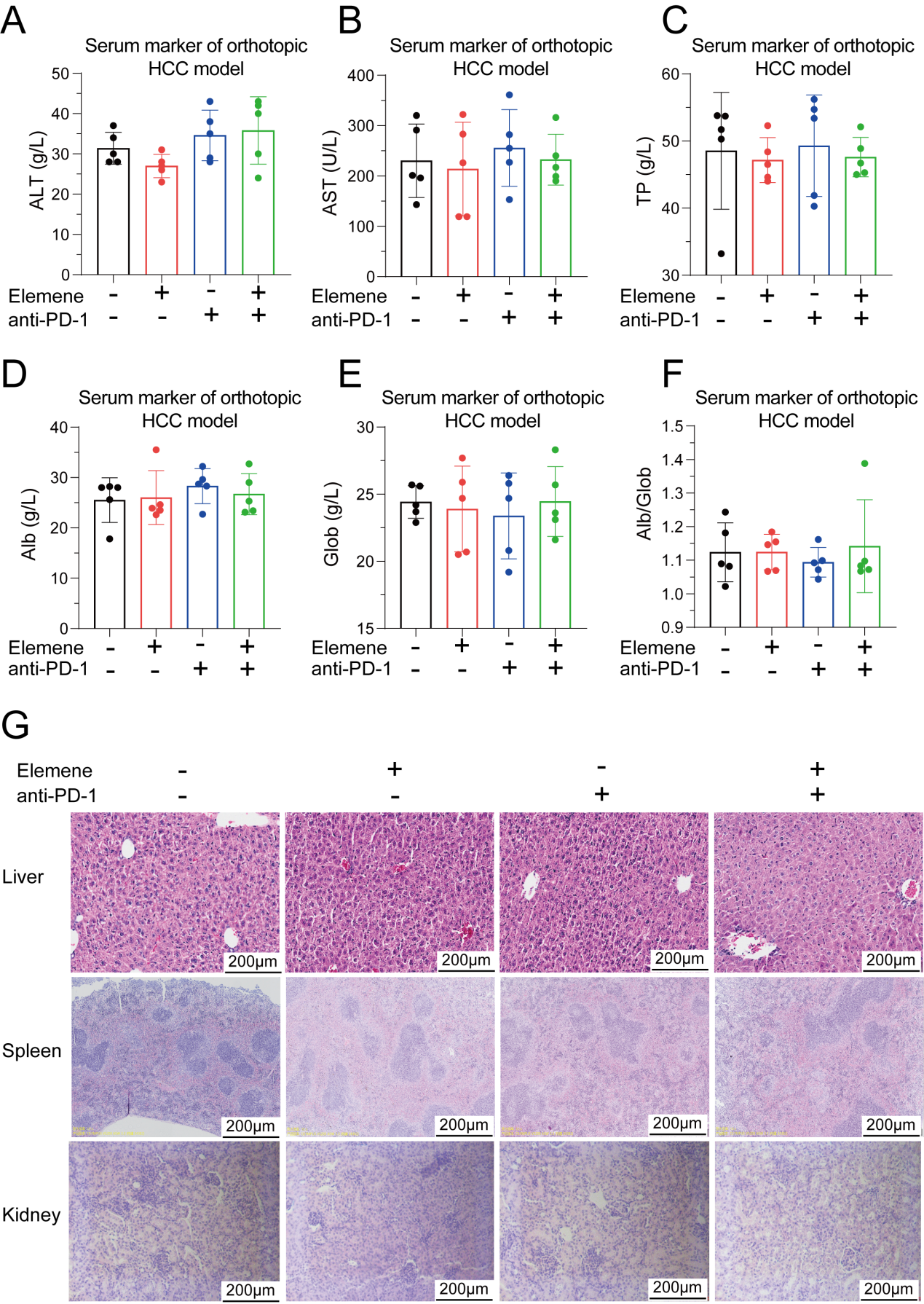


**Figure S1.** **EP treatment did not result in remarkable toxicity in orthotopic HCC model.** (**A**-**F)** Serum levels of markers for liver function, including ALT (A), AST (B), TP (C), Alb (D), and Glob (E) and the Alb/Glob ratio (F), in the orthotopic HCC model treated with the indicated regimens. n = 5. The data are presented as the means ± SD. **(G)** Representative H&E-stained images of livers, spleens and kidneys from orthotopic HCC model treated with the indicated regimens. Scale bar = 200 μm.


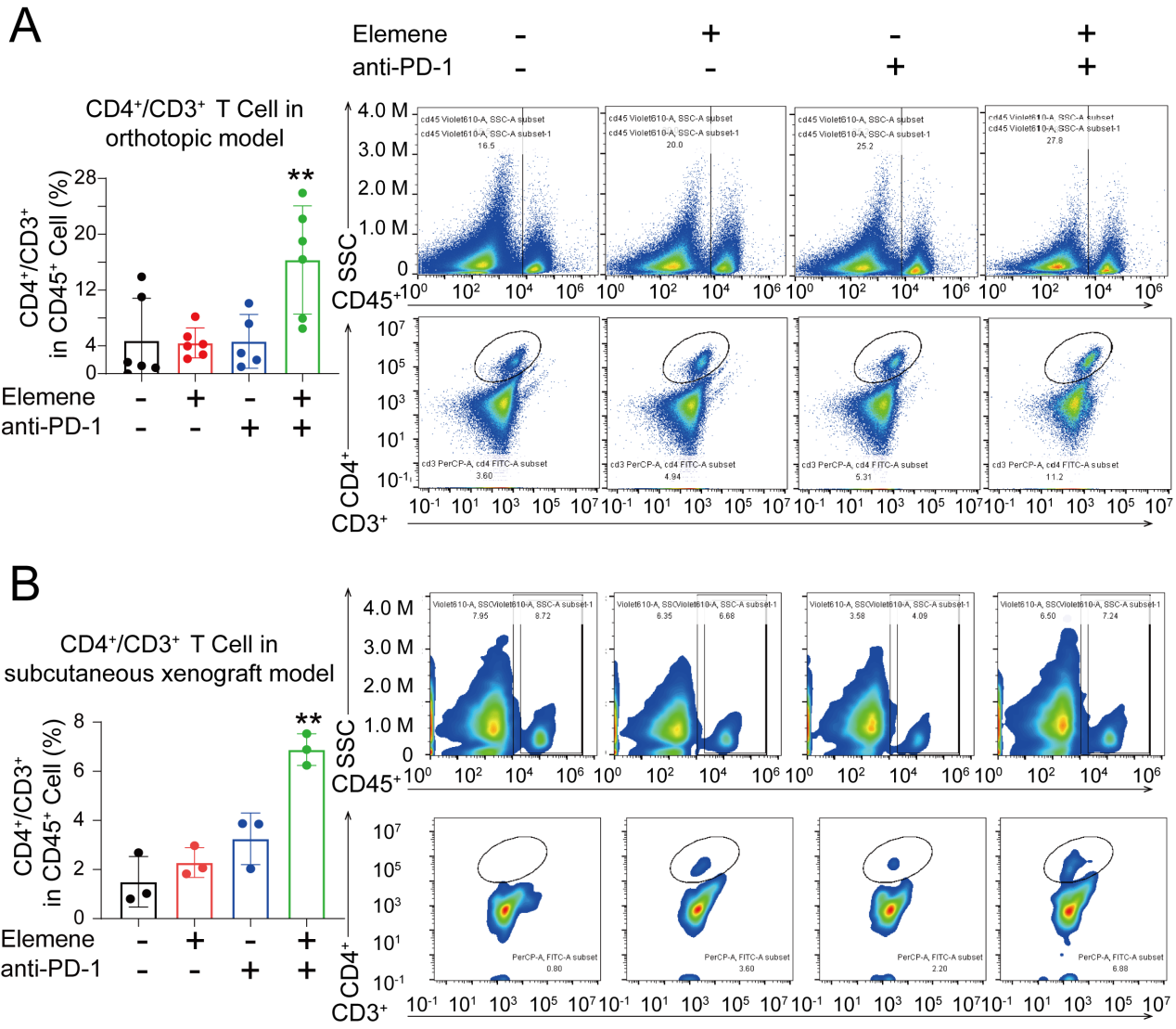


**Figure S2. Intratumoral CD4^+^ T cells infiltration was enhanced by EP therapy in the murine HCC models. (A)** Histogram and representative flow cytometry images showing CD4^+^/CD3^+^ T cells among CD45^+^ lymphocytes in tumor tissues of orthotopic HCC model treated with the indicated regimens. n = 6. The data are expressed as the means ± SEM. **(B)** Histogram and representative flow cytometry images of CD4^+^/CD3^+^ T cells among CD45^+^ lymphocytes in tumor tissues of subcutaneous xenograft HCC model treated with the indicated regimens. n = 3. The data are presented as the means ± SEM. *^**^P* < 0.01.


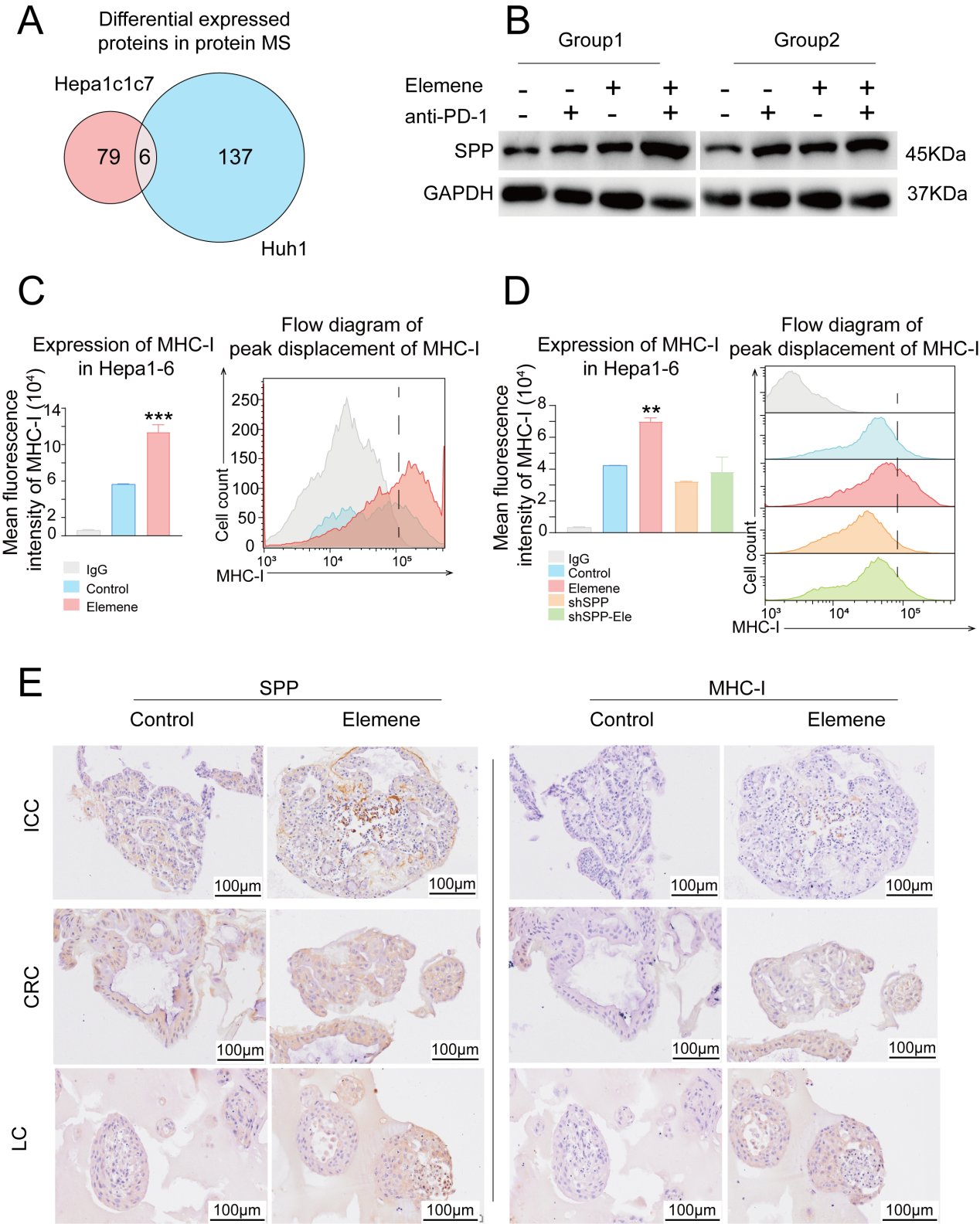


**Figure S3. Elemene increased the abundance of MHC-I/peptide complexes in HCC cells by enhancing SPP expression. (A)** Venn diagram illustrating the number of significantly regulated proteins identified in Hepa1c1c7 and Huh1 cells following elemene treatment. (|Log2FC| > 0.5, *P* < 0.05). **(B)** Western blots showing SPP protein expression in two sets of tumor tissues from orthotopic HCC model. **(C)** Quantitative analysis of the mean fluorescence intensity and representative flow cytometry histogram of MHC-I expression in Hepa1-6 cells after elemene treatment. n = 3. The data are presented as the means ± SD. **(D)** Quantitative analysis of the mean fluorescence intensity and representative flow cytometry histogram showing the change in MHC-I expression after elemene treatment in Hepa1-6 cells transfected with shscramble or shSPP. n = 3. The data are presented as the means ± SD. **(E)** Representative IHC images showing the expression of SPP and MHC-I in patient-derived organoids of various cancer types treated with elemene, including intrahepatic cholangiocarcinoma (ICC), and colorectal cancer (CRC), LC. Scale bar = 100 μm. *^**^P* < 0.01, *^***^P* < 0.001.

**
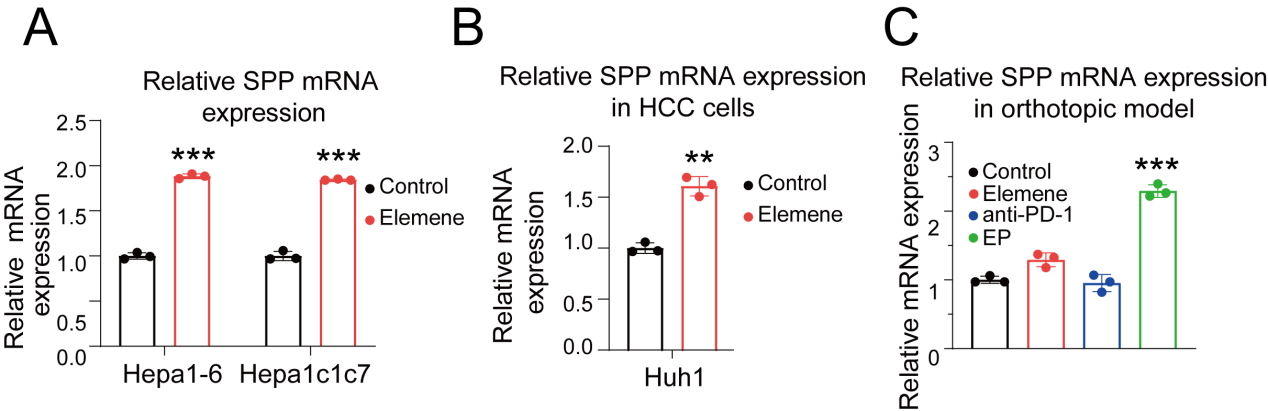
**

**Figure S4. Elemene enhanced the SPP mRNA expression. (A**, **B)** SPP mRNA expression in the murine HCC cell lines Hepa1-6 and Hepa1c1c7 (A) and the human HCC cell lines Huh1 (B) after treatment with elemene. n=3. The data are presented as the means ± SD. Student’s t test was used for analysis. (**C)** Expression level of intratumoral SPP mRNA after the indicated treatment in the orthotopic HCC model. n=3. The data are presented as the means ± SD. Student’s t test was performed to compare each treatment group to the control group. ^***^*P* < 0.001.

**
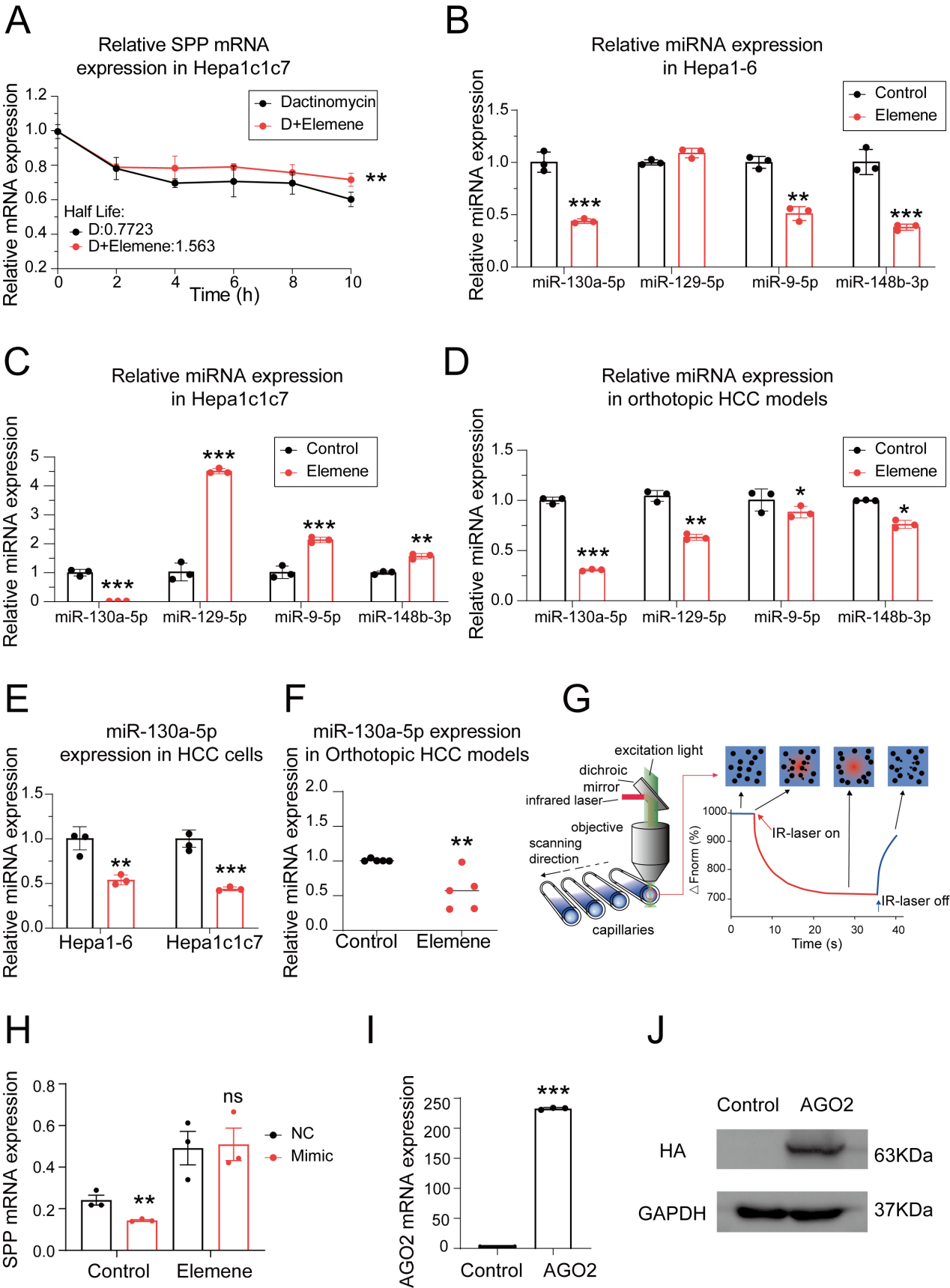
**

**Figure S5. Elemene inhibited the degradation of SPP mRNA and down-regulated the expression of miR-130a-5p. (A)** Degradation curves of SPP mRNA in Hepa1c1c7 cells following actinomycin D treatment and elemene pretreatment. Nonlinear regression curve fitting (one-phase decay) was used to calculate the decay rates. n = 3. The data are presented as the means ± SEM. **(B**-**D)** Expression levels of 4 candidate miRNAs in Hepa1-6 (B), Hepa1c1c7 (C), and tumor tissues from the orthotopic HCC model (D) after elemene treatment. n = 3. The data are presented as the means ± SD. **(E)** Relative miR-130a-5p expression levels in Hepa1-6 and Hepa1c1c7 cells receiving elemene treatment. n = 3. **(F)** Relative miR-130a-5p expression levels in the tumor tissues of xenograft HCC model receiving elemene treatment. n = 5. The data are presented as the means ± SD. **(G)** Schematic of the microscale thermophoresis (MST) assay for detecting molecular interactions between elemene and miR-130a-5p or control miRNA (miRNA-6353). **(H)** Relative SPP mRNA levels in HEK293T cells pre-treated with miR-130a-5p mimic in the presence or absence of elemene. n = 3. The data are presented as the means ± SD. **(I)** Expression AGO2 mRNA levels in HEK 293T cells. n = 3. The data are presented as the means ± SD. **(J)** Expression of HA-tagged AGO2 protein in HEK293T cells. *^*^P* < 0.05, *^**^P* < 0.01, *^***^P* < 0.001.

**
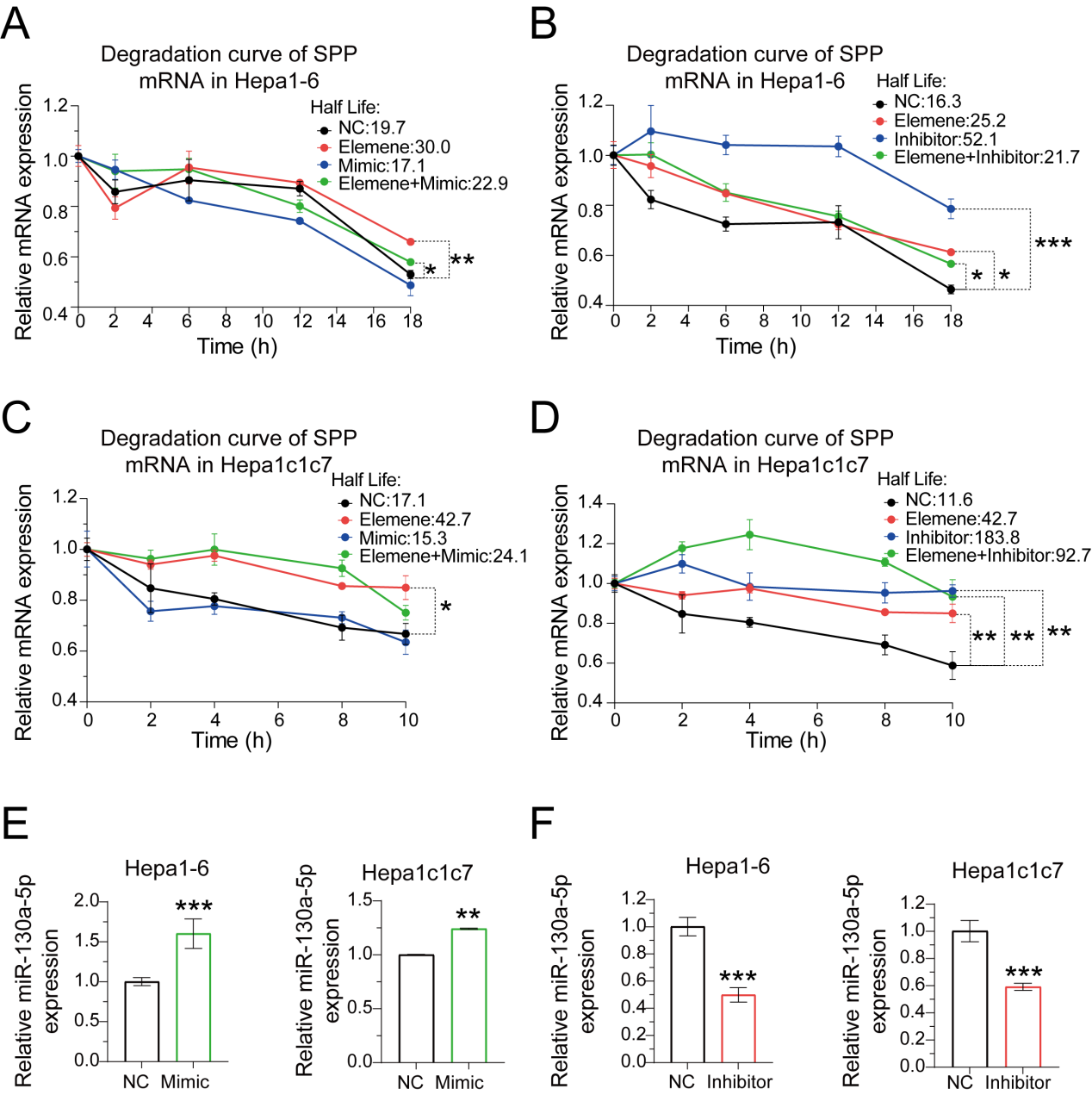
**

**Figure S6. Elemene enhanced SPP expression by directly competing with miR-130a-5p.** **(A**, **B)** Degradation curves of SPP mRNA in Hepa1-6 cells pre-treated with elemene following actinomycin D treatment in the presence or absence of the miR-130a-5p mimic (A) and inhibitor (B). n = 3. The data are presented as the means ± SEM. **(C**, **D)** Degradation curves of SPP mRNA in Hepa1c1c7 cells pre-treated with elemene following actinomycin D treatment in the presence or absence of the miR-130a-5p mimic (C) and inhibitor (D). n = 3. The data are presented as the means ± SEM. **(E)** Expression levels of miR-130a-5p in Hepa1-6 and Hepa1c1c7 cells treated with the miR-130a-5p mimic. n = 3. The data are presented as the means ± SD. **(F)** Expression levels of miR-130a-5p in Hepa1-6 and Hepa1c1c7 cells treated with the miR-130a-5p inhibitor. n = 3. The data are presented as the means ± SD. *^*^P* < 0.05, *^**^P* < 0.01, *^***^P* < 0.001.


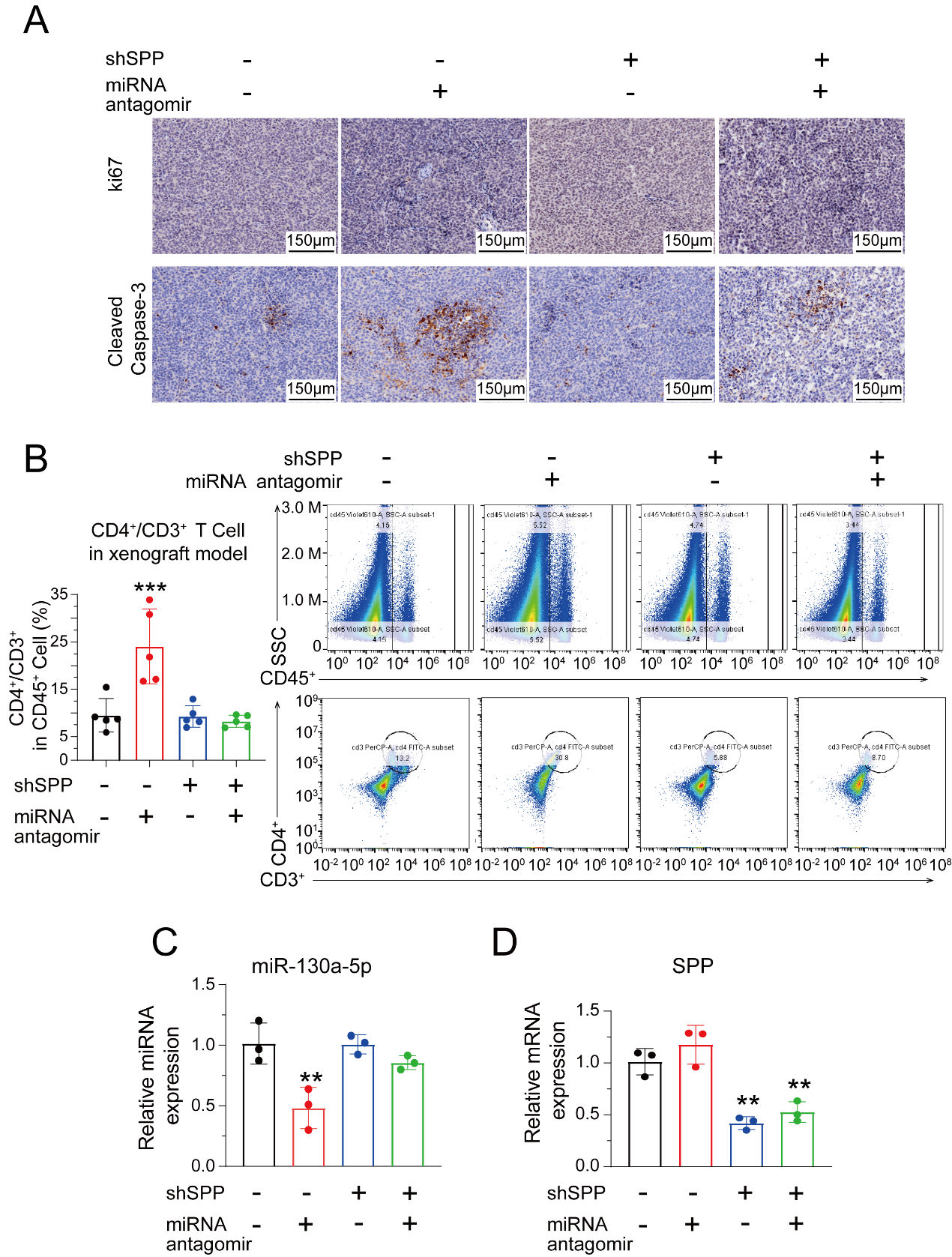


**Figure S7.** **The inhibition of miR-130a-5p significantly delayed tumor growth and promoted immune infiltration via SPP.** **(A)** Representative IHC images of Ki-67 and cleaved caspase-3 in tumor tissues from mice bearing Hepa1-6-shscramble and Hepa1-6-shSPP xenografts after treatment with the miR-130a-5p antagomir. Scale bar = 150 μm. **(B)** Histogram and representative flow cytometry images of CD4^+^/CD3^+^ T cells among CD45^+^ lymphocytes in the tumor tissues of subcutaneous xenograft model. n = 5. The data are presented as the means ± SD. **(C, D)** Expression levels of miR-130a-5p (C) and SPP mRNA (D) in the tumor tissues of the subcutaneous xenograft model. n = 3. The data are presented as the means ± SD. *^**^P* < 0.01, *^***^P* < 0.001.


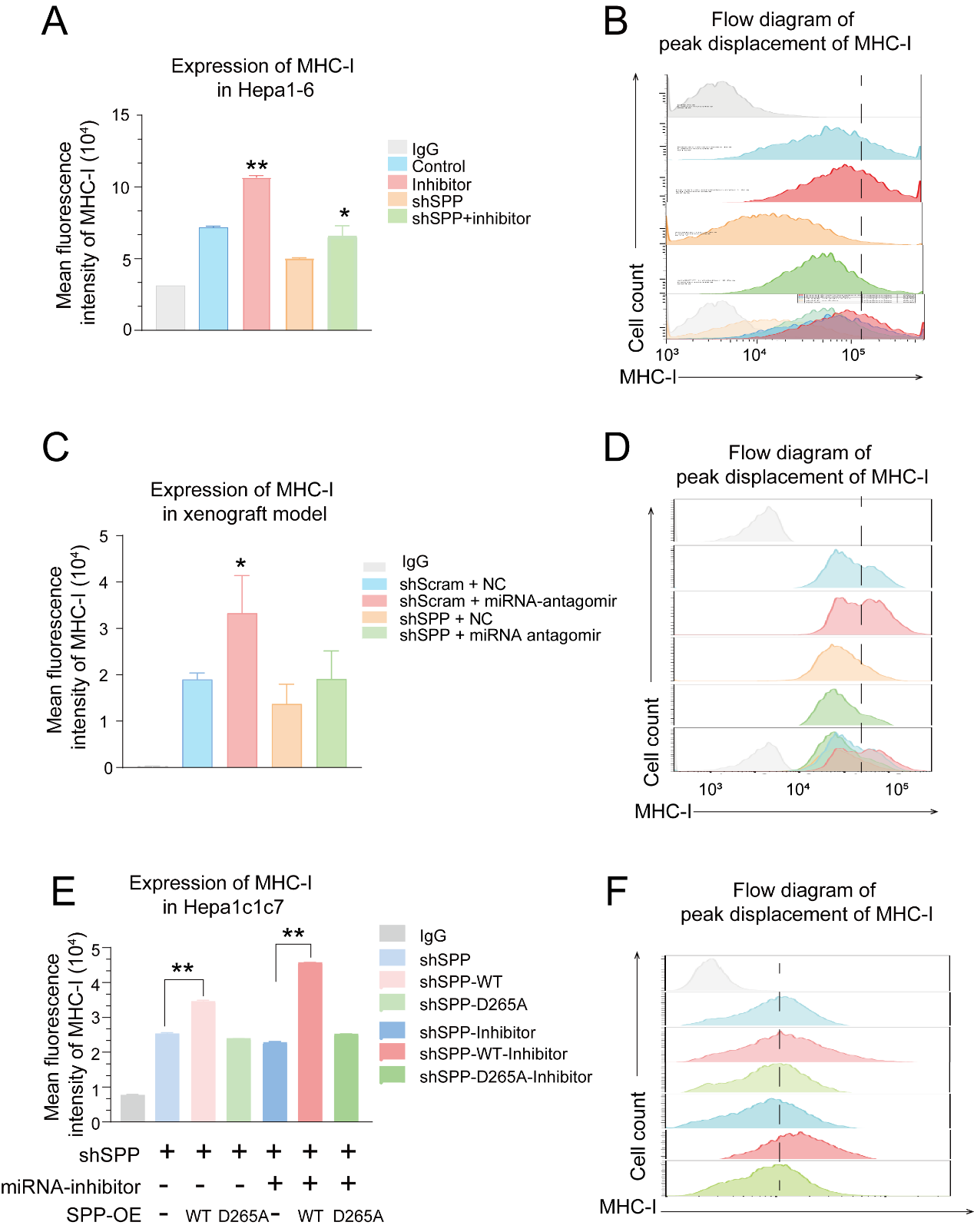


**Figure S8.** **miR-130a-5p could regulate the abundance of membrane MHC-I via SPP.** (**A**, **B**) Quantitative analysis of the mean fluorescence intensity (A) and representative flow cytometry histogram (B) of MHC-I expression in Hepa1-6 cells transfected with shscramble or shSPP in the presence or absence of the miR-130a-5p inhibitor. n = 3. The data are presented as the means ± SD. Student’s t test was used to compare the inhibitor and control groups for each cell type. **(C, D)** Quantitative analysis of the mean fluorescence intensity (C) and representative flow cytometry histogram (D) of MHC-I expression in Hepa1-6 cells transfected with shscramble or shSPP in the presence or absence of the miR-130a-5p antagomir. n = 6. The data are presented as the means ± SD. Student’s t test was used to compare the inhibitor and control groups for each cell type. **(E)** Surface MHC-I expression was quantified as mean fluorescence intensity from flow cytometry data. n = 3. The data are presented as the means ± SD. Student’s t test was used to compare the inhibitor and control groups for each cell type. **(F)** Representative flow cytometry histograms showing surface MHC-I expression levels across the indicated experimental groups in SPP-knockdown (shSPP) HCC cells. ^*^*P <* 0.05*, ^**^P <* 0.01*.*


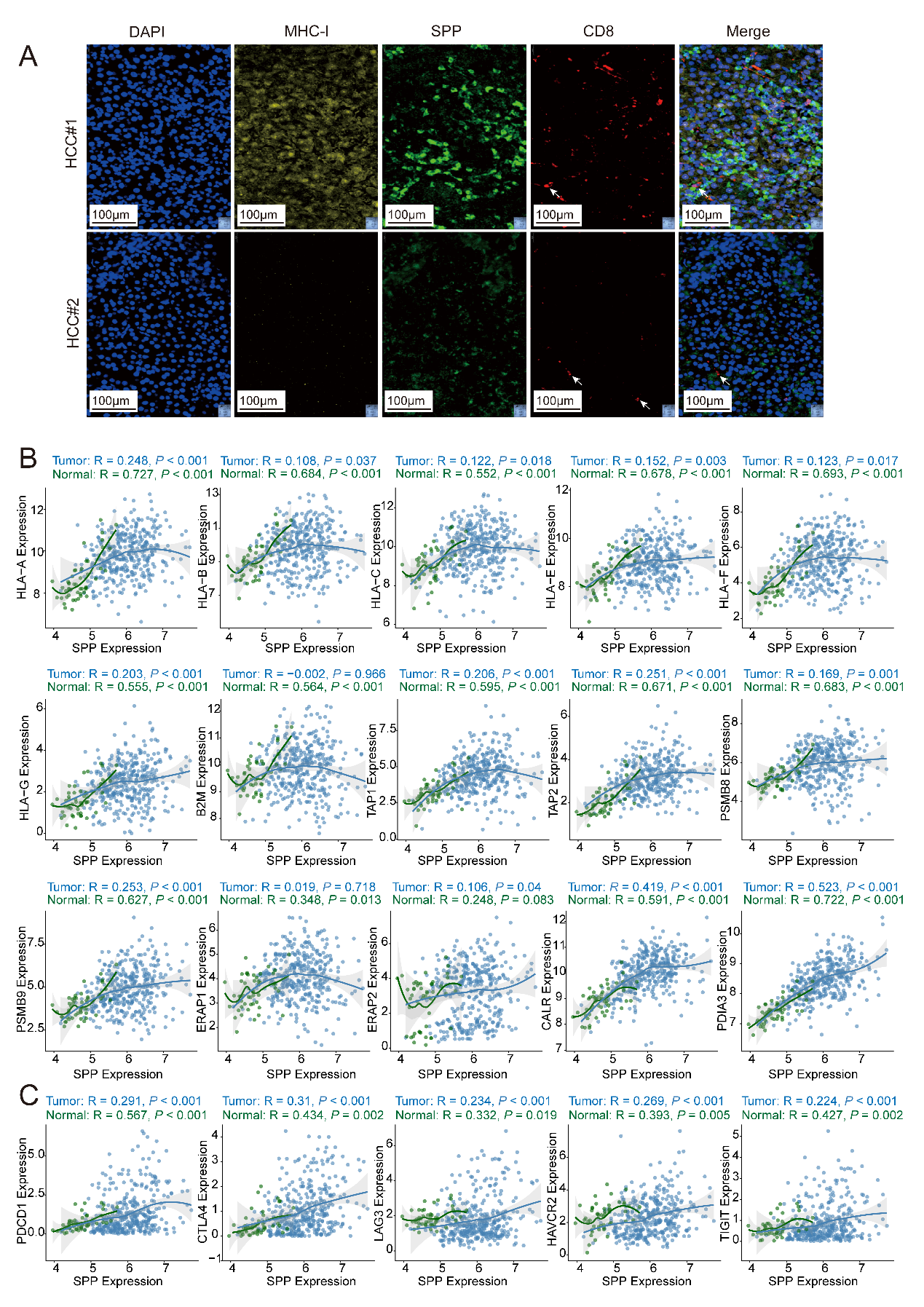


**Figure S9. Clinical association of SPP expression with immune cell infiltration in HCC. (A)** Representative multiplex immunofluorescence (mIF) images of MHC-I (yellow), SPP (green), and CD8 (red) in human HCC samples. Nuclei are counterstained with DAPI (blue). Scale bar = 100 μm. **(B)** Scatter plots showing the correlations between SPP expression and the mRNA levels of individual key genes constituting the MHC-I antigen presentation complex in the TCGA-LIHC cohort. **(C)** Scatter plots showing the correlations between the expression levels of SPP and key immune checkpoint genes in the TCGA-LIHC cohort.


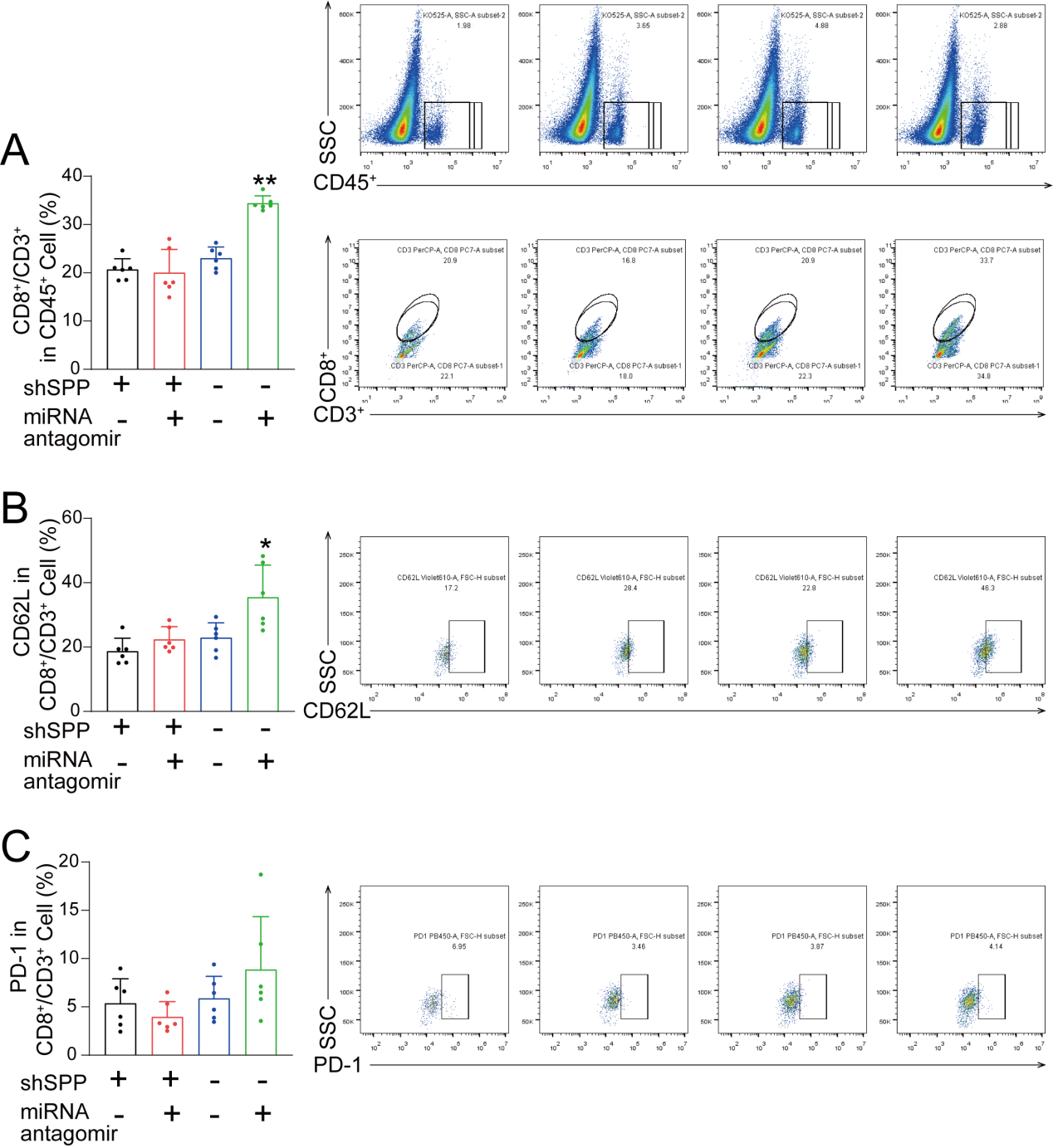


**Figure S10. (A)**Histogram of the percentage and representative images of flow cytometry analysis on CD8^+^/CD3^+^ T expression of CD45^+^ lymphocytes in tumor tissues of subcutaneous xenograft HCC model. n = 6. The data are presented as the means ± SEM. **(B)** Histogram of the percentage and representative images of flow cytometry analysis on T-cell memory (CD62L) expression of CD8^+^/CD3^+^ T-cell in tumor tissues of subcutaneous xenograft HCC model. n = 6. The data are presented as the means ± SEM. **(C)** Histogram of the percentage and representative images of flow cytometry analysis on T-cell exhaustion (PD-1) expression of CD8^+^/CD3^+^ T-cell in tumor tissues of subcutaneous xenograft HCC model. n = 6. The data are presented as the means ± SEM. **P <* 0.05*, **P <* 0.01*.*


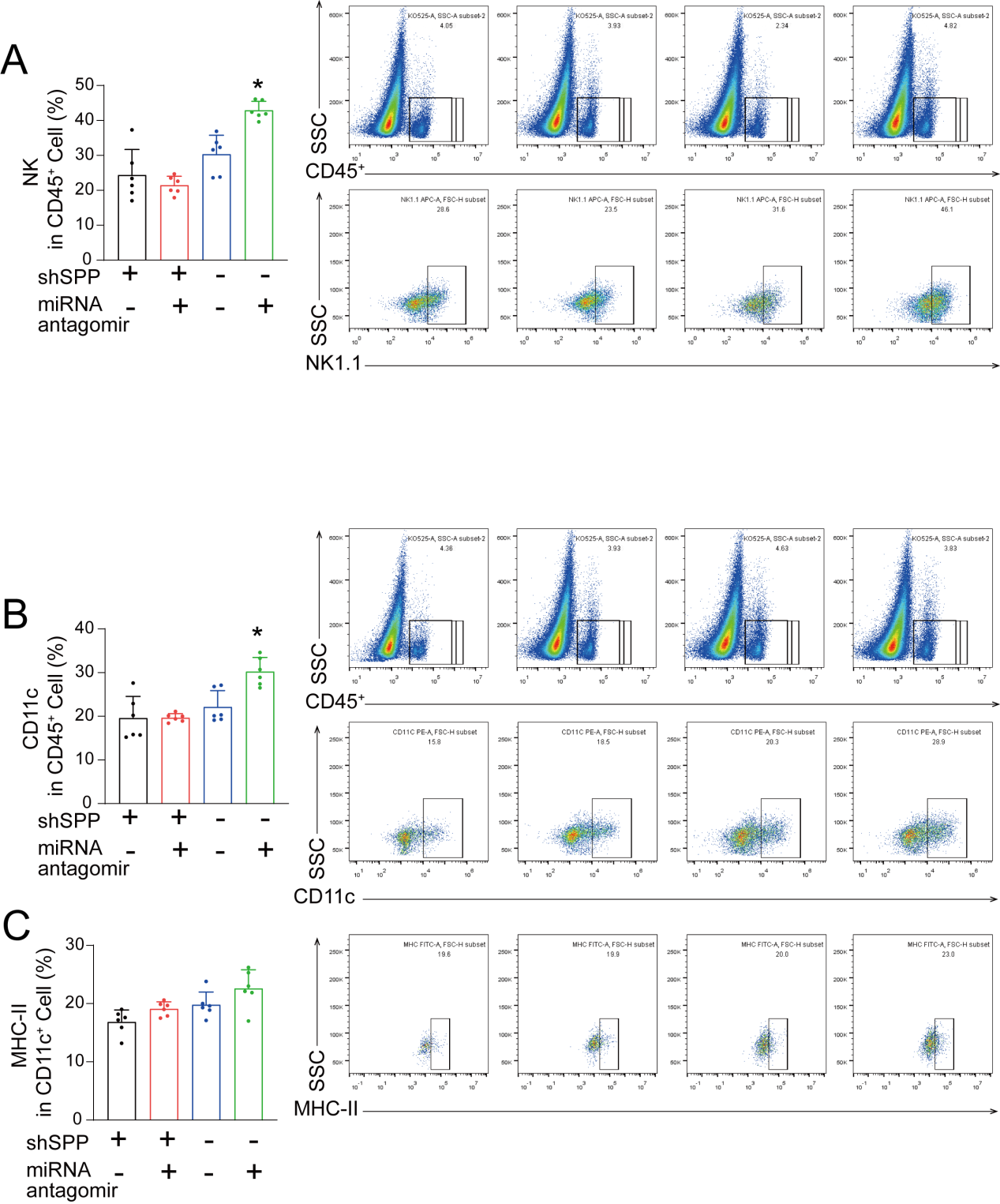


**Figure S11. (A)** Histogram of the percentage and representative images of flow cytometry analysis on NK cell (NK1.1) expression of CD45+ lymphocytes in tumor tissues of subcutaneous xenograft HCC model. n = 6. The data are presented as the means ± SEM. **(B)** Histogram of the percentage and representative images of flow cytometry analysis on DC cell (CD11c) expression of CD45+ lymphocytes in tumor tissues of subcutaneous xenograft HCC model. n = 6. The data are presented as the means ± SEM. **(C)** Histogram of the percentage and representative images of flow cytometry analysis on MHC-II expression of CD11c+ DC in tumor tissues of subcutaneous xenograft HCC model. n = 6. The data are presented as the means ± SEM.**P* < 0.05.


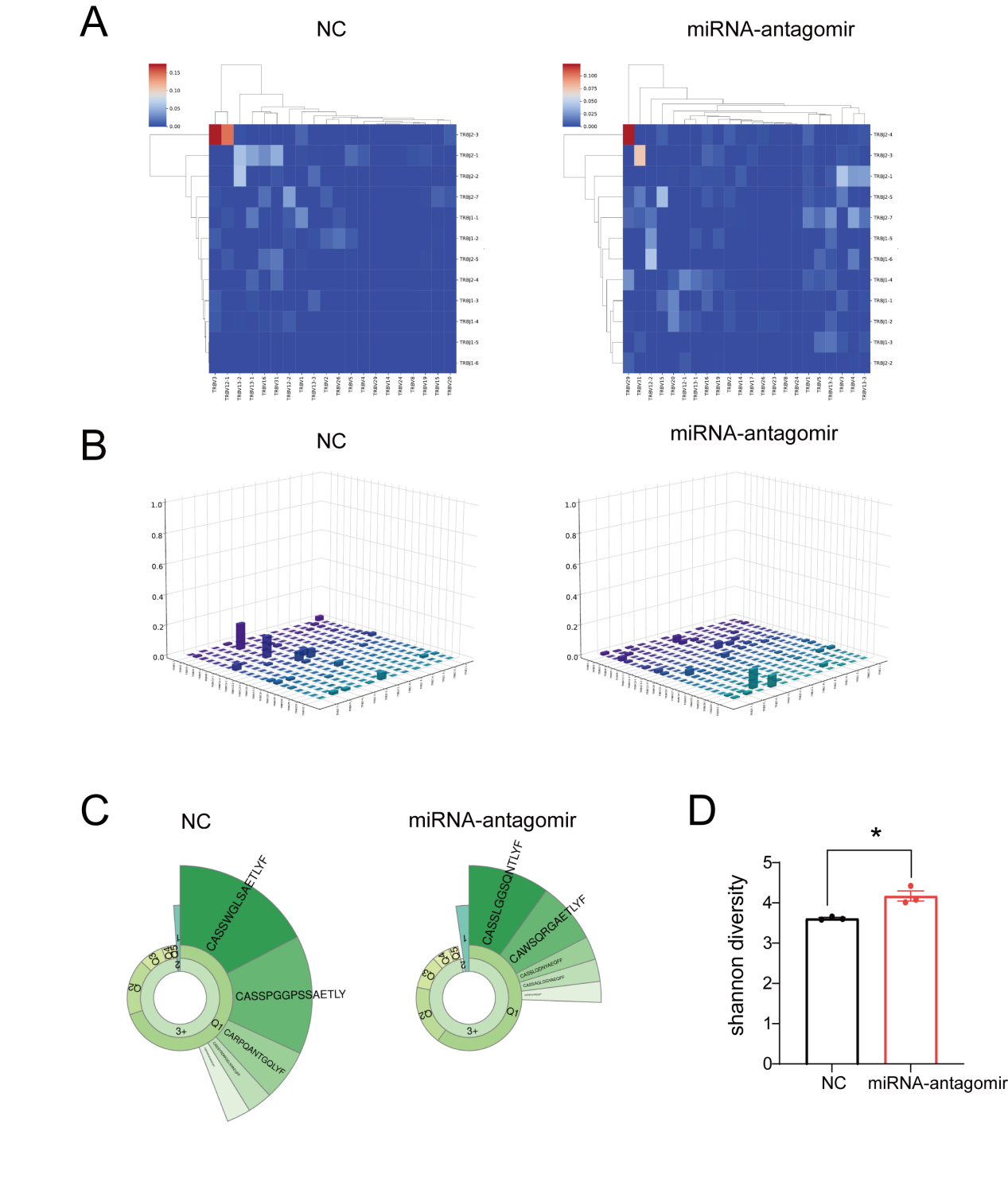


**Figure S12. miRNA-antagomir increased CTL diversity during HCC immunotherapy. (A, B)** Heatmaps (A) and 3D forest maps (B) showing the frequency of each TCR-β chain V--J fragment combination in the tumor tissues of subcutaneous xenograft HCC model after the indicated treatment. The high-, moderate- and low-frequency combinations in the heatmaps are colored red, white and blue, respectively. The frequency of each V--J fragment combination is shown as the height of the bar in the 3D forest maps. **(C)** Snail plots showing the classification of TCR-β chain clonal frequencies in tumor tissues of the subcutaneous xenograft HCC model after the indicated treatments. Categorizes all detected TCR sequences into "1", "2", and "3+" groups, representing TCR molecules detected once, twice or more than 3 times in the TCR sequencing assay, respectively. The "1" category consists of a large number of distinct T-cell clones, whose TCR sequences were only detected once during the TCR sequencing assay. These low-abundance clones are precisely the basis of clonal polymorphism. **(D)** Comparison of T cell clonal diversity based on Shannon diversity index. The Shannon diversity index was calculated from TCR sequencing data to quantitatively evaluate the diversity of the T cell repertoire across different treatment groups. n = 3. The data are presented as the means ± SD. **P* < 0.05.

**Supplementary tables**

**Table S1. KPS improvement in tumor patients treated with Elemene**

| **Study** | **Disease** | **Basic treatment** | **Elemene group** | | **Control group** | |
| --- | --- | --- | --- | --- | --- | --- |
|  |  |  | Events | Total | Events | Total |
| Tian L^[1]^ | NSCLC | Chemotherapy | 16 | 32 | 7 | 30 |
| Yang Z^[2]^ | NSCLC | Chemotherapy | 36 | 60 | 23 | 60 |
| Wang L^[3]^ | NSCLC | Chemotherapy | 20 | 30 | 12 | 30 |
| Cong L^[4]^ | NSCLC | Radiotherapy | 7 | 25 | 2 | 25 |
| Hao S^[5]^ | NSCLC | Chemotherapy | 15 | 27 | 7 | 27 |
| Wang H^[6]^ | NSCLC | Chemotherapy | 20 | 31 | 11 | 30 |
| Ou P^[7]^ | NSCLC | Chemotherapy | 15 | 25 | 6 | 21 |
| Song F^[8]^ | NSCLC | Chemotherapy | 40 | 60 | 24 | 60 |
| Zhang Y^[9]^ | NSCLC | Chemotherapy | 20 | 40 | 11 | 40 |
| Zhou Y^[10]^ | NSCLC | Targeted therapy | 39 | 60 | 15 | 60 |
| Wei Q^[11]^ | GC | Chemotherapy | 24 | 30 | 16 | 30 |
| Qin Z^[12]^ | GC | Chemotherapy | 15 | 34 | 6 | 34 |
| Zeng D^[13]^ | GC | Chemotherapy | 12 | 25 | 6 | 24 |
| Yu D^[14]^ | GC | Chemotherapy | 16 | 34 | 6 | 31 |
| Qu S^[15]^ | GC | Chemotherapy | 11 | 34 | 5 | 34 |
| Zhu B^[16]^ | GC | Chemotherapy | 17 | 32 | 10 | 32 |
| He J^[17]^ | GC | Chemotherapy | 13 | 40 | 7 | 40 |
| Liang T^[18]^ | MTB | Radiotherapy | 13 | 30 | 4 | 30 |
| Guo Y^[19]^ | MTB | Radiotherapy | 12 | 18 | 7 | 18 |
| Tian F^[20]^ | MTB | Radiotherapy | 23 | 42 | 13 | 42 |
| Cheng H^[21]^ | EC | Chemotherapy | 10 | 18 | 8 | 18 |
| Song Z^[22]^ | EC | Radiotherapy | 16 | 30 | 7 | 30 |
| Lan T^[23]^ | LC | Targeted therapy | 14 | 36 | 10 | 37 |
| Wang M^[24]^ | LC | Chemotherapy | 14 | 30 | 6 | 30 |

Abbreviations: Karnofsky Performance Scale (KPS), Non-small cell lung cancer (NSCLC), Gastric cancer (GC), Metastatic tumor of brain (MTB), Esophageal carcinoma (EC), Liver cancer (LC).

**Table S2. KPS improvement rate in tumor patients treated with Elemene**

| **Disease** | **Study** | **n1/N1*** | **n2/N2*** | **Heterogeneity test** | **RR**  **(95%CI)** | ***P*** |
| --- | --- | --- | --- | --- | --- | --- |
| NSCLC | 10 | 228/390 | 118/383 | P=0.878, I2 =0% | 1.90  (1.60-2.25) | <0.001 |
| GC | 7 | 108/229 | 56/225 | P=0.869, I2 =0% | 1.90  (1.47-2.45) | <0.001 |
| MTB | 3 | 48/90 | 24/90 | P=0.517, I2=0% | 2.00  (1.36-2.95) | <0.001 |
| EC | 2 | 26/48 | 15/48 | P=0.222, I2 =33% | 1.73  (1.06-2.84) | 0.029 |
| LC | 2 | 28/66 | 16/67 | P=0.367, I2=0% | 1.78  (1.06-2.97) | 0.028 |

*n1(n2), the number of cases with improved KPS in the Elemene group (Control group). N1 (N2), the total number of cases in the Elemene group (Control group).

**Table S3. The sequences of primers, siRNAs, Mimic, inhibitor, antagomir**

| Identifier | Sequences (5'-3') |
| --- | --- |
| Mouse SPP Forward | GGGCTCTGGGGAAAACAAAG |
| Mouse SPP Reverse | CAAAGGACTTGGCCACTGTC |
| Human SPP Forward | CACGCCCGAGGGCATCGCGC |
| Human SPP Reverse | GTCCATGTATTTCTTCGTGC |
| Human GAPDH Forward | CTGCCAACGTGTCAGTGGTG |
| Human GAPDH Reverse | TCAGTGTAGCCCAGGATGCC |
| Mouse GAPDH Forward | AGAAGGTGGTGAAGCAGGCATC |
| Mouse GAPDH Reverse | CGAAGGTGGAAGAGTGGGAGTTG |
| U6 Forward | GCTTCGGCAGCACATATACTAAAAT |
| U6 Reverse | CGCTTCACGAATTTGCGTGTCAT |
| miR-148b-3p Forward | CGCGTCAGTGCATCACAGAA |
| miR-148b-3p Reverse | AGTGCAGGGTCCGAGGTATT |
| miR-130a-5p Forward | CGCGGCTCTTTTCACATTGT |
| miR-130a-5p Reverse | AGTGCAGGGTCCGAGGTATT |
| miR-129-5p Forward | CGCTTTTTGCGGTCTGG |
| miR-129-5p Reverse | AGTGCAGGGTCCGAGGTATT |
| miR-9-5p Forward | GCGCGTCTTTGGTTATCTAGCT |
| miR-9-5p Reverse | AGTGCAGGGTCCGAGGTATT |
| miR-148b-3p Reverse Transcription Primer | GTCGTATCCAGTGCAGGGTCCGAGGTATTCGCACTGGATACGACACAAAG |
| miR-130a-5p Reverse Transcription Primer | GTCGTATCCAGTGCAGGGTCCGAGGTATTCGCACTGGATACGACAGTAGC |
| miR-129-5p Reverse Transcription Primer | GTCGTATCCAGTGCAGGGTCCGAGGTATTCGCACTGGATACGACGCAAGC |
| miR-9-5p Reverse Transcription Primer | GTCGTATCCAGTGCAGGGTCCGAGGTATTCGCACTGGATACGACTCATAC |
| Mouse si-SPP-1 | CACCAUCUUCAUCAUGCACAU |
| Mouse si-SPP-2 | GAGAUCAUCAACUAUGAGUUU |
| Mouse miR-130a mimics | GCUCUUUUCACAUUGUGCUACU |
| Mouse miR-130a inhibitor | AGUAGCACAAUGUGAAAAGAGC |
| miR-130a-5p antagomir | (mA)*(mG)*(mU)(mA)(mG)(mC)(mA)(mC)(mA)(mA)(mU)(mG)(mU)(mG)(mA)(mA)(mA)(mA)*(mG)*(mA)*(mG)*(mC) |
| Control antagomir | (mU)*(mC)*(mU)(mA)(mC)(mU)(mC)(mU)(mU)(mU)(mC)(mU)(mA)(mG)(mG)(mA)(mG)(mG)(mU)*(mU)*(mG)*(mU)(mG)*(mA) |

References

1. Tian L, Cao Y, Peng Z, Wang W, Long N. Elemene emulsion combined with chemotherapy in the treatment of patients with advanced non-small cell lung cancer (Chinese). *Chin J Clin Med*. 2009;16:725-727.
2. Yang Z, Wang L. Clinical research of combined elemene for injection with DP therapy in treating elderly patient with advanced non-small cell lung cancer (Chinese). *J Tradit Chin Med Univ Hunan*. 2009;29:5-9.
3. Wang L, Chen F. Elemene emulsion combined with NP chemotherapy in the treatment of advanced NSCLC patients (Chinese). *Chin J Cancer Res*. 2010;20:547-550.
4. Cong L. *Clinical study on radiotherapy combined with elemene emulsion in the treatment of elderly patients with non-small cell lung cancer* (Chinese). Master's thesis. Beijing University of Chinese Medicine; 2012.
5. Hao S, Zhang J, Zang T. Observation of the synergistic attenuating effect of elemene emulsion on platinum-based chemotherapy regimens in the treatment of advanced non-small cell lung cancer (Chinese). *Clin Focus*. 2012;27:1529-1531.
6. Wang H, Wu X, Li L. Clinical efficacy of elemene injection combined with paclitaxel and carboplatin in the treatment of advanced non-small cell lung cancer (Chinese). *J Mod Oncol*. 2012;20:978-980.
7. Ou P, An Y. Comparison study of elemene combined with temozolomide, temozolomide alone maintenance therapy after radiotherapy in patients of NSCLC with brain metastases (Chinese). *Med Philos*. 2014;35:29-32.
8. Song F, Song F, Li T. Elemene combined with NP chemotherapy in the treatment of advanced non-small-cell lung carcinoma (Chinese). *Med Recapitulate*. 2015;21:1710-1712.
9. Zhang Y, Li W, Hu X. Elemene combined with TC chemotherapy in the treatment of advanced non-small-cell lung cancer (Chinese). *China Pract Med*. 2015;10:173-175.
10. Zhou Y, Wan D, Qian X, Jian H, Feng J. Effect of elemene injection combined with gefitinib on immune function and life quality of advanced elder lung cancer patients with EGFR mutant (Chinese). *Chin J Clin Healthc*. 2017;20:502-505.
11. Wei Q, Shou J. Study on therapeutic effects of treating progressive gastric cancer with elemene emulsion combined with fluorouracil/paclitaxel (Chinese). *Zhejiang Clin Med J*. 2008;05:588-589.
12. Qin Z, Lu L, Yuan G, et al. Elemene combined olf program of treatment of advanced gastric cancer (Chinese). *J Tradit Chin Med*. 2010;28:435-437.
13. Zeng D, Bi Y, Ling Y, Yang Q. Clinical research of elemene emulsion combined with FOLFOX 4 regimen for advanced gastric cancer (Chinese). *J Clin Oncol*. 2011;16:917-919.
14. Yu D, An G, Dai H. Efficacy observation of advanced gastric cancer treated with elemene and tegafur (Chinese). *World J Integr Tradit West Med*. 2013;8:264-266.
15. Qu S, Liu Y, Liu Z, et al. Elemene oral emulsion combined with S-1 in the treatment of elderly advanced gastric cancer (Chinese). *J Mod Oncol*. 2017;25:2280-2283.
16. Zhu B. Efficacy and safety of elemene combined with chemotherapy in gastrointestinal tumors (Chinese). *J North Pharm*. 2017;14:157.
17. He J, Xie J, Chen G. Efficacy of oxaliplatin combined with elemene oral milk in the treatment of 40 elderly patients with advanced gastric cancer (Chinese). *J Clin Med Pract*. 2015;19:131-132.
18. Liang T. *Clinical study on elemene injection combined with radiotherapy in the treatment of non-small cell lung cancer with brain metastasis* (Chinese). Master's thesis. Beijing University of Chinese Medicine; 2011.
19. Guo Y, Suo D. Clinical observation on elemene injection combined with radiotherapy in the treatment of advanced lung cancer with brain metastasis (Chinese). *Chin Community Doctors*. 2015;31:88-89.
20. Tian F, Du J. To observe the clinical curative effect of patients with elemene injection adjuvant treatment of brain metastases of lung cancer (Chinese). *Chin J Hosp Pharm*. 2016;36:395-397.
21. Cheng H, Yang Z, Zhang M, Chen Z. Clinical observation of elemene combined with paclitaxel/tegafur in the treatment of advanced esophageal carcinoma (Chinese). *Anhui Med Pharm J*. 2012;16:1679-1681.
22. Song Z. Efficacy of elemene combined with systemic Gamma Knife in the treatment of advanced esophageal cancer (Chinese). *Shandong Med J*. 2013;53:62-63.
23. Lan T, Chen C. Effect of elemene on patients with advanced liver cancer of leptin, AFP and liver function (Chinese). *Chin J Biochem Pharm*. 2015;35:116-118.
24. Wang M, Qian Y, Qian C, Zhang H, Yang W, Jiang H. Clinical study on Lianqi capsule combined with elemene injection on patients with middle-late primary liver cancer and its effects on serum growth factor (Chinese). *Hebei J Tradit Chin Med*. 2015;40:234-240.
